# Supplementary material for: MinION™ nanopore sequencing of environmental metagenomes: a synthetic approach
Source: Gigascience. 2017 Feb 24;6(3):1–10. doi: 10.1093/gigascience/gix007 (PMC5467020; doi:10.1093/gigascience/gix007)
Supplement: GIGA-D-16-00081_Original_Submission.pdf [file gix007_GIGA-D-16-00081_Original_Submission.pdf]

Manuscript

Click here to view linked References

Click here to download Manuscript GigaScience SynthMetagenom 11viii16.docx

MinION™ nanopore sequencing of environmental metagenomes: a synthetic approach

Bonnie L. Brown, Virginia Commonwealth University, Department of Biology, 1000 W Cary Street, Richmond, VA 23284, USA, blbrown@vcu.edu

Mick Watson, The Roslin Institute, University of Edinburgh, Division of Genetics and Genomics, Easter Bush, Midlothian, EH25 9RG, UK, mick.watson@roslin.ed.ac.uk

Kevin P. Keegan, Argonne National Laboratory, Biosciences Division, 9700 South Cass Avenue, Argonne, IL 60439, USA, kkeegan@anl.gov

Maria C. Rivera, Virginia Commonwealth University, Department of Biology, 1000 W Cary Street, Richmond, Virginia 23284, USA, mcrivera@vcu.edu

Rima B. Franklin, Virginia Commonwealth University, Department of Biology, 1000 W Cary Street, Richmond, Virginia 23284, USA, rbfranklin@vcu.edu

Corresponding author: Bonnie L. Brown, blbrown@vcu.edu

Abstract

Background: Environmental metagenomics is typically accomplished by annotating WGS sequences or assigning taxonomy based on 16S amplicon sequences. Both of these approaches provide species annotations that are limited by read length. A nanopore-based sequencing platform, MinION™, produces reads that are $\geq 1 \times 10^4$ bp in length, potentially providing for more precise assignment of taxa, thereby alleviating some of the error inherent in determining metagenome composition from short reads. We tested the ability of sequence data produced by MinION (R7.3 flow cells) to correctly classify prokaryotes in single-species runs and in three types of simple mixed synthetic communities: a mixture of DNA using equal quantities from four species, a community with one relatively rare (1%) and three common (33% each) components, and a community with 20 species of staggered representation. Community analysis was performed by three methods: BlastN, Kraken, and MG-RAST.

Results: MinION long reads for single-species runs (R7.3 flow cells) yielded as few as 224 to as many as 3,497 bidirectional high-quality (2D) reads with an average overall study length of 6,000 bp. For the single-species analyses, assignment of reads to the correct genus ranged from 25% to 95%, and the majority of mis-assigned reads were to related organisms. The synthetic metagenome yielded 714 high quality 2D reads of approximately 5,500 bp that were 88% correctly assigned. Synthetic metagenomes run using the MinION Mk1 device (R7.3 flow cells) yielded 899-3,497 2D reads with lengths averaging 5,700 bp; the best assignment being 97% correct. Community proportions for “equal” and “rare” synthetic libraries were close to the known proportions, deviating from 0.1 – 10% across all tests. For a 20-species mock community with staggered contributions, a sequencing run detected all but 3 species (each included at <0.05% of DNA in the total mixture); 71% of reads were assigned to the correct species, 75% of reads were assigned to the correct genus, and >99% of reads were assigned to the correct family.

Conclusions: At the present high quality output level (just under 4×10^3 2D pass reads for an environmental metagenome), MinION sequencing followed by Kraken analysis has the potential

to provide rapid and accurate metagenomic analysis where the environmental consortium is comprised of a limited number of taxa. Important considerations noted in this study are that the MinION platform is very sensitive to DNA quality, variation is high across libraries and flow cells, the relatively small numbers of 2D reads per analysis limit metagenomic analysis where thousands of taxa are expected, and these same qualities limit detection of very rare components of microbial consortia. Nevertheless, the fact that >97% of high quality reads generated by MinION can be confidently assigned to expected taxa and in most cases mirror the known proportions in a mixture warrants further exploration of practical application to environmental metagenomics as the platform continues to develop and improve. With advances in throughput and error rate, this platform shows great promise for precise real-time analysis of both the components and the relative representation of more complex communities, free from amplification biases introduced by PCR-based metagenomic analyses.

Keywords

MinION™, Oxford Nanopore Technologies, metagenome, whole-genome sequencing, long-read sequencing

Background

Environmental metagenomics employs molecular genetic markers to identify ecologically and epidemiologically important components of sediments, soils, waters, and surfaces. Knowledge of the consortia that inhabit these ecosystems allows for better understanding of the organisms and their ecological roles, provides for creation of effective strategies to mitigate ecosystem damage, and allows evaluation of the responses of species to environmental change. One common sequencing approach in environmental metagenomics involves sequencing and subsequent annotation of whole genome nucleic acid fragments (WGS) extracted directly from water or sediment to discover major components of the ecosystem; if sequenced deeply enough, rare species can be detected. For well-studied components of the microbial community, such metagenomic data also can be used to characterize the functional potential of complex communities.

One approach to analyzing a set of WGS sequences is to assemble them into large contigs prior to analysis and annotation. If assembled correctly, contigs have a greater chance of identifying the true components; however, when assembling contigs from a community of related species, there is a high likelihood of generating chimeric contigs. If WGS reads are not assembled, there is no chance of chimeras and more true diversity can potentially be uncovered through annotation, but very short reads can lack the information needed to properly identify the source of the read due to conservation of gene sequence across related organisms. The high information content of very long reads such as those provided by MinION™ (Oxford Nanopore Technologies, Inc., Oxford, UK) has the potential to overcome some of the limitations of short reads by allowing for longer alignments that potentially can contribute to higher taxonomic specificity and resolution. Although conceived almost two decades ago [1], nanopore-based whole-molecule sequencing has only recently become available to MinION™ Access Programme (MAP) participants for exploration and practical application [2]. Data generated by early access MinION™ flow cells have been assessed for whole genome sequencing [3, 4, 5, 6, 7], gene expression and transcriptome studies [8, 9, 10], clinical applications such as antibiotic resistance, influenza, and Ebola [11, 12, 13], and bacterial and viral serotyping [14]. One study has evaluated the potential of this new sequencing platform to address clinical metagenomes of viral pathogens [15]; however, the MinION has not to date been evaluated for the potential characterization

of environmental metagenomes. To demonstrate the potential of this platform for broader applications, we performed a set of experiments to quantify the ability of MinION™ long-read sequence data to accurately determine the structure of metagenomes by assessing its performance in identifying the taxonomy and relative proportions of the organisms in synthetic metagenomes.

Data description

The raw MinION data [16] collected during sequencing by MinKNOW software (versions 0.49.2.9 through 0.51.3.40 b201605171140) were immediately uploaded as FAST5 packets to Metrichor Agent (r7.3 2D basecalling, ver rx-2.22-44717-dg-1.6.1-ch-1.6.3; Mk1 2D basecalling, ver WIMP Bacteria k24 for SQK-MAP006), after which base-called data [16] were returned to the host computer, also in the form of FAST5 files. The programs poRe [17], Poretools [18], and NanoOK [19] were used to extract and characterize the numbers of reads and channels, archive only the 2D reads into a FASTQ and FASTA files for downstream analyses. The basecalled data sets were scrutinized by methods commonly employed in metagenome analysis including WIMP [20], BlastN [21] versus a database of all bacteria, Kraken [22] which indexed more than 5000 genomes and plasmids, MG-RAST [23] which assigned taxa based on predicted proteins and rRNA genes, and principal components analysis based on the frequency of 5-mers in each read then annotated with the top BlastN hit, and this was carried out in R [24].

Results

MinION™ WGS libraries were generated from 1 μg of fresh DNA isolates (see Methods) of laboratory cultures of two Proteobacteria, *Escherichia coli* and *Pseudomonas fluorescens*, and two Cyanobacteria, *Microcystis aeruginosa*, and *Synechococcus elongatus*, and from mixtures of these four species (either equivalent amounts of DNA or using three common and one rare species), yielding sufficient Pre-sequencing Mix for multiple loads of each flow cell. An additional library prepared from a commercially prepared 20-species mock community required genome pre-

amplification using Φ29 polymerase to generate sufficient material to prepare the sequencing library because only 100 ng of material are provided by the supplier of this product.

The single-species runs generated up to 22×10^3 reads ($0.2–1.1\times10^3$ 2D reads) ranging from as short as 5 bp to as long as 267×10^3 bp (Table 1), of which ≥90% of assignments for the high quality 2D reads for *E. coli*, *P. fluorescens,* and *S. elongatus* concurred with the known species (Table 2). For *M. aeruginosa*, at best 58% of 2D reads were correctly assigned to the level of species, although many of the mis-assigned reads aligned to closely related cyanobacteria genera, Sphingobacteriaceae, and other prokaryotes known to break down microcystin [25]. Comparative 16S analysis of the various preparations used to construct the synthetic communities confirmed identity of the former three strains, whereas for the latter strain *M. aeruginosa*, the 16S analysis indicated 90% similarity to *M. aeruginosa* and *M. panniformis* (Table 3).

In the second round of validation, using three synthetic communities containing mixtures of the same afore-described species, $6–12\times10^3$ reads ($0.7–1.3\times10^3$ 2D reads) were generated per run, ranging in length from $0.6\text{-}56.8\times10^3$ bp (Table 1). For the two communities comprised of equal DNA contribution from four eubacteria (25% each species), WGS proportions aligned roughly but not exactly with the known proportions (Table 2). In the early run (MinION with R7.3 flow cell) specifically, classification results were 27% *E. coli*, 16% *M. aeruginosa,* 30% *P. fluorescens,* 21% *S. elongatus*, 3% Enterobacteriaceae, and 3% misclassified. In the subsequent test (Mk1 with R7.3 flow cell), classification results were 26% *E. coli*, 18% *M. aeruginosa*, 30% *P. fluorescens*, 22% *S. elongatus,* and 3% Enterobacteriaceae, and 1% misclassified (Figure 1). For the community with three common (33% of each) and one rare (1%) representative, classifications were 33% *E. coli*, 34% *P. fluorescens,* 29% *S. elongatus,* 1% *M. aeurginosa,* 2% misclassified (a third of those latter category of reads were assigned to *Shigella*). Read annotation of 5-mers for both the "Equal" and "Rare" community data sets using the top BlastN hit for each read revealed that 5-mer frequency profiles of MinION long-read sequences were shared within species. This was reflected in the 5-mer frequency analysis which revealed distinct clusters in the PCA (Figure 2).

In the final round of testing, the mock microbial community with 20 species included in "staggered" proportions (i.e., 1,000 to 1,000,000 16S rRNA operon copies per organism per μL of material supplied) yielded 14.7×10^3 reads (3.5×10^3 2D reads) ranging in length from $0.5\text{-}20.9 \times 10^3$ bp, sufficient to detect all of the high and moderate species, but the sequencing run failed to detect 3 of 5 species that were included at very low rRNA operon copy number (Table 4). For that run, 25% of reads were to Enterobacteriaceae and misclassifications accounted for only 0.2% of read assignments. Greatly overrepresented in the results for this run were reads assigned to *E. coli* (included as 20% of DNA but observed as 46% of read assignments), whereas greatly underrepresented in the results were reads assigned to *R. sphaeroides* which was included as 41% of DNA but accounted for only 1% of read assignments (Figure 3).

Table 1 MinION™ sequencing output details for single-species and synthetic mixtures.

Experiment (chemistry)	Pores with reads	Run time (hr)	Total bp (Gbp)	Total Reads	Number of 2D reads	Mean 2D read length (bp)	MG-RAST Accession
E. coli (5)	430	42	83.6	26590	1112	5274	4629367.3
P. fluorescens (5)	453	48	119.4	25228	777	7784	4629445.3
M. aeruginosa (5)	377	18	40.8	22760	569	5676	4629369.3
S. elongatus (5)	367	23	18.3	6163	224	5101	4629381.3
Equal mixture (25% of each of four species)							
(5)	129	24	26.5	10592	714	5527	4614572.3
(6)	437	44	77.1	12174	1358	5202	4685746.3
Rare mixture (1% of *M. aeruginosa* and 33% each of three other species)							
(6)	449	18	39.0	6728	899	6194	4685745.3
Staggered mixture (20 species in various proportions)							
(6)	300	33	39.0	14711	3497	2612	4705090.3

The four single species were sequenced using the original MinION device with SQK–MAP005 chemistry: indicated by (5). The equal mixture was sequenced using both the original MinION device with SQK–MAP005 chemistry and the Mk1 (SQK-MAP006 chemistry: indicated by (6)). Columns relating to 2D indicate bi-directional reads with quality above Q9.

Table 2 Assignment of metagenomic reads based on three analyses.

Experiment	BLASTN			KRAKEN			MG-RAST		
	**Known**	**Mis**	**Un**	**Known**	**Mis**	**Un**	**Known**	**Mis**	**Un**
E. coli	95.0	4.2	0.8	90.9[a]	0.5	1.7	50.3[a]	31.0[b]	18.3
P. fluorescens	79.3	16.6	4.1	79.3	15.2	5.5	79.5	19.6	0.9
S. elongatus	82.8	2.4	14.8	91.5	1.8	6.7	85.9	12.6	1.5
M. aeruginosa	24.8	7.4	67.8	45.0	8.9	46.1	58.4[a]	35.0	5.5
Equal mixture (25% each of four spp)									
(5)	88.2[a]	4.6	7.1	76.1	11.6	12.3	61.4	38.5[c]	0.1
(6)	93.5[a]	2.7	3.7	95.4	2.0	2.6	77.1	22.6[b]	0.3
Rare mixture (1% of *M. aeruginosa* and 33% each of three other spp)									
(6)	96.6	1.0	2.4	95.2	0.9	3.9	79.6	20.2[b]	0.2
Staggered mixture (20 species in various proportions)									
(6)	92.1	3.6[b]	4.3	89.3	1.9	8.8	54.1	28.8[b]	17.1

Percent of 2D reads assigned to the known genus (Known), assigned to another genus (Mis), and unclassified at the level of genus (Un) for single-species DNA preparations (all run using the original MinION™ platform) and synthetic mixtures sequenced on the MinION and analyzed by MG-RAST, Kraken, and BlastN. [a]In some cases, reads were classified only to a higher (correct) taxonomic level than genus, resulting in totals < 100% for various analyses. [b]Mostly *Shigella*. [c]Mostly *Stentrophomonas* and *Shigella*.

Table 3 Breakdown of BlastN assignment of 16S reads for single-species DNA preparations used in this study.

Culture	Final sequence Length (bp)	%	Sequence matches in BlastN Organism
Escherichia coli	1440	97	*E. coli* numerous strains
Escherichia coli	1696	99	*E. coli* numerous strains
Escherichia coli	1604	99	*E. coli* numerous strains
Microcystis aeruginosa	1418	90	*M. aeruginosa* NIES-843 and NIEHS-2549, and *M. panniformis* FACHB-1757
Pseudomonas fluorescens	1570	96	*P. fluorescens* A506 and LBUM223
Pseudomonas fluorescens	1386	96	*P. fluorescens* A506 and LBUM223
Synechococcus elongatus	1719	99	*S. elongatus* PCC 7942, PCC 6301, UTEX 2973
Synechococcus elongatus	1640	99	*S. elongatus* PCC 7942, PCC 6301, UTEX 2973
Synechococcus elongatus	1642	99	*S. elongatus* PCC 7942, PCC 6301, UTEX 2973
Synechococcus elongatus	1431	99	*S. elongatus* PCC 7942, PCC 6301, UTEX 2973
Synechococcus elongatus	1583	99	*S. elongatus* PCC 7942, PCC 6301, UTEX 2973

Table 4 Known and estimated composition of 20-species mock staggered community.

**Organism**	**Operon count/µL**	**Quantity pg/µL**	**% DNA in template**	**WIMP % species**	**WIMP % Genus**
Acinetobacter baumannii	10,000	8.2	0.24	0.14	0.14
Actinomyces odontolyticus	1,000	1.0	0.03	nd	nd
Bacillus cereus	100,000	45	1.33	0.53	0.53
Bacteroides vulgatus	1,000	0.8	0.02	0.10	0.10
Clostridium beijerinckii	100,000	44	1.30	0.19	0.19
Deinococcus radiodurans	1,000	1.0	0.03	0.05	0.05
Enterococcus faecalis	1,000	0.7	0.02	nd	nd
Escherichia coli	1,000,000	680.0	20.04	45.61	45.66
Helicobacter pylori	10,000	8.6	0.25	1.68	1.68
Lactobacillus gasseri	10,000	3.2	0.09	0.14	0.14
Listeria monocytogenes	10,000	5.0	0.15	0.38	0.38
Neisseria meningitides	10,000	5.8	0.17	0.24	0.24
Propionibacterium acnes	10,000	8.8	0.26	0.48	0.48
Pseudomonas aeruginosa	100,000	160.0	4.71	1.25	1.25
Rhodobacter sphaeroides	1,000,000	1,400.0	41.25	1.01	1.01
Staphylococcus aureus	100,000	59.0	1.74	0.38	3.88
Staphylococcus epidermidis	1,000,000	510.0	15.03	7.67	7.72
Streptococcus agalactiae	100,000	32.0	0.94	0.96	1.01
Streptococcus mutans	1,000,000	420.0	12.38	10.17	10.17
Streptococcus pneumoniae	1,000	0.6	0.02	nd	nd
Other[a,b]		0.0	0.00	29.02[a]	25.37[b]
Correct assignments				70.98	74.63

Prokaryote DNA included in the 20-species mock staggered community (obtained from bei Resources, ATCC, HM-783D, provided at 5.5 ng/µL, pre-amplified with Φ29 polymerase prior to MinION™ sequencing) compared with WIMP analysis result. “nd”: not detected. [a]Of these, 12.7% were correctly assigned to genus, 86.4% were Enterobacteriaceae, and only 0.7% were misclassifications. [b]Of these, 86.4% were Enterobacteriaceae and only 0.7% were misclassified.

Discussion

Sequencing of whole genome preparations can enhance environmental metagenomic analysis by providing more precise identification of species diversity [26, 27]. Typical environmental samples contain tens of thousands to millions of organisms, yet the resulting metagenomes almost certainly underrepresent this diversity, and often due to short-read strategy, the resulting data sets can be confidently assigned only to higher taxonomic levels [28, 29]. One strategy to alleviate this problem is to carefully assemble metagenomic data, which despite the resulting chimerism has been shown to greatly enhance species call correctness [30]. However, even with enhanced sequencing and bioinformatic strategies, many public database accessions contain extra-species sequences, e.g., symbionts, parasites, pathogens, and sequencing linkers/primers/adapters (unbeknownst to those who have accessed the data) that can lead to false discovery rates [31]. Hybrid accessions in particular, can potentially affect metagenome results predicated on short reads to a greater extent that would be expected from analyses based on long reads, which have the potential to circumvent the effect of hybrid accessions. Long reads can circumvent these issues assuming that much of the genome for each component organism is represented in the sequencing library and that there are few errors in the sequences and the comparative database. The results reported here allow us to consider the potential for long read sequencing via MinION and subsequent metagenomic analysis. We found that for low complexity synthetic communities, the results indicate that long reads provided by MinION R7.3 flow cells provide sufficiently precise sequence data to assign groups represented at or above 1%. In fact, 2 of 5 species included at <0.05% in a mock community were detected. Furthermore, for un-amplified whole genome preparations, read assignments were observed to be within about 10% of their proportional occurrence in the metagenome. Ultimately, we saw that although the reads were longer, because the sequence coverage was not as deep, the improvement in specificity of assignment was offset by a reduction in the sensitivity, and some of the genomes present at low concentration were not sequenced.

The fact that the estimated proportions of community members were not precise despite careful quantitation could indicate differences across library preparation (all libraries were prepared

by BLB), reagent kits, flow cells, MinKNOW control scripts, the quality of DNAs used to create the synthetic metagenomes, and the methods used for quantification (Qubit for the home-grown mixtures and UV spectrophotometry for the 20-species mixture). Because DNA quality is of paramount importance for MinION sequencing, PreCR (SQK-MAP005 protocol) or FFPE Repair Mix (SQK-MAP006 protocol) was included in the preparation of all libraries. The potential for profound effects related to library preparation recently was examined [27] leading to the recommendation that studies of complex metagenomes should be based on PCR-free approaches. The current data indicate that the MinION lends itself well to a PCR-free approach but that utility for analysis of complex metagenomes is limited at the current time by the small number of reads that pass the quality filtering process. The current study also provides data for considering alternatives to PCR for amplification, in this case GenomiPhi™, which was used to generate sufficient DNA for one library in the current study ("Staggered"). This method is optimized for linear DNA and was intended to generate unbiased copies of the 20-species genomes. Nevertheless, this pre-amplification step is one likely reason that could account for the overrepresentation of *E. coli* and underrepresentation of *R. sphaeroides* in the Staggered run. However, another distinct possibility that could account for incongruence of known and estimated proportions in the Staggered community is that organisms for which there are many accessions in the public databases provide for more precise classification (e.g., *E. coli* complete genome accessions) and that *vice versa*, organisms with relatively few accessions (e.g., *R. sphaeroides* accessions) result in less precise classification. A third possibility is that the original DNA preparation for *R. sphaeroides* was less pure or more degraded than preparations for the other 19 species. A novel low input DNA approach recently reported [32] could enhance such MinION analyses.

Despite the rather small number of 2D reads that were observed to pass the quality filter across all MinION runs, there was a strong biological signal in the data (Figure 2). Thus, as investigators have found MinION useful for single genome introspection [4, 7, 13], 16S and other amplicon resolution [14, 33], cDNA sequencing [9], and assembly [3, 34, 35], our findings imply that this platform has immediate utility for analysis of very simple mixtures (e.g., serum testing for

pathogens). Over the 18-month period of MinION use for this set of experiments, 2D pass rates increased from 2% to 24%. That rate of improvement is concurrent with Moore's Law [36] and lends support to the expectation that the platform will continue to improve for the foreseeable future. The cloud-based WIMP base-calling and taxon prediction program associated with the device provides a method of real-time analysis of metagenomic data. However, because we had no control over the comparative database, the cloud implementation of WIMP was far less flexible for environmental metagenomic analysis than local Kraken, and we note that use of an inappropriate or incomplete database can lead to false positives and negatives. Further investigations of MinION output and data analysis should focus on evaluation of the informative value of 1D reads, alternative alignment algorithms, and more sophisticated k-mer analyses. As the quality rate for this platform improves, the potential will increase for MinION to provide data for the majority of organisms that are included in an environmental metagenome.

Methods

To set a baseline of expectations for MinION metagenomic analysis, we performed single-species sequencing runs with four organisms. Cell cultures at log phase were harvested by spinning 15 mL culture tubes at 3,000 × g for 30 min and DNA was isolated using the PowerSoil DNA kit (MoBio, Carlsbad, CA, USA) according to the manufacturer's instructions. Nucleic acid quality and quantity were checked via Nanodrop 2000 and Qubit, whereafter 1 μg of DNA was used to prepare sequencing libraries. For the first two mixtures, equal portions of DNAs from all four organisms (250 ng each) were used ("equal") and for the third mixture ("rare"), equivalent amounts of three of the species were used (330 ng each) and *M. aeruginosa* was included as only 1% of the mixture (10 ng). An additional preparation of a mock community containing 20 microbe species was obtained from a commercial source (HM-783D, bei Resources, ATCC, Manassas, VA, USA). Because sequencing libraries for this study began with 1 μg of DNA, to generate sufficient starting material 1 μL of the mock community sample (5.5 ng of template) was pre-amplified using Φ29 enzyme from the GenomiPhi

V3 kit (25-6601-24, GE Healthcare Bio-Sciences, Pittsburgh, PA, USA) according to the manufacturer's recommendation.

Sequencing libraries were prepared for R7.3 flow cells run on an original MinION device using the Genomic DNA Sequencing Kit SQK–MAP005 according to the base protocol from Oxford Nanopore with slight modifications [37] and for R7.3 flow cells run on the Mk1 device using the Nanopore Sequencing Kit SQK–MAP006 according to the manufacturer's recommendations. The steps for library SQK–MAP005 preparation included in this order: shearing 1 μg in a Covaris g-TUBE (Covaris, Inc., Woburn, MA, USA) at 2,000 × g for 2 min, treatment with PreCR (New England Biolabs, Beverly, MA, USA), cleanup with 1X AMPure beads (Agencourt, Beckman Coulter, Brea CA, USA), end-repair with NEBNext End Repair Module (New England Biolabs), cleanup with 0.5X AMPure beads, dA-tailing with NEBNext dA-Tailing Module (New England Biolabs), ligation to a cocktail of both the leader and hairpin sequencing adapters (Oxford Nanopore Technologies) using Blunt TA Ligase (New England Biolabs), cleanup using his-tag Dynabeads (Life Technologies, City, State, USA), and recovery of the pre-sequencing mix in 25 □L of Elution Buffer (Oxford Nanopore Technologies). After priming the flow cell with EP solution according to the manufacturer's recommendations, an initial 6 μL aliquot of the pre-sequencing mix (at 10-20 ng/μL) was combined with 141 μL EP Solution and 3 μL Fuel Mix and applied to the flow cell. Thereafter, at 6-8 hr intervals, additional pre-sequencing mix aliquots (held on ice) combined with EP Solution and Fuel Mix were added to the flow cell at times roughly coinciding with re-programmed pore "remux," which is a process that adjusts the bias voltage and mux channels to maximize yield performance. Modified scripts (J. Tyson, pers. comm.) caused the MinION device to perform four remux steps at 8 h intervals to maintain regular increases in data (Figure 4).

Steps for library SQK–MAP006 preparation included in this order: shearing in a Covaris g-TUBE (Covaris, Inc., Woburn, MA, USA) at 2,000 × g for 2 min, treatment with PreCR (New England Biolabs, Beverly, MA, USA), cleanup with 1X AMPure beads (Agencourt, Beckman Coulter, Brea CA, USA), combined end-repair and dA-tailing with NEBNext UltraII End Repair/dA-Tailing Module (New England Biolabs), cleanup with 1X AMPure beads, ligation to a cocktail of both the

leader and hairpin sequencing adapters (Oxford Nanopore Technologies) using Blunt TA Ligase (New England Biolabs), addition of a tether to the hairpin segment, cleanup using MyOne Streptavidin C1 Beads (Life Technologies, Carlsbad, CA, USA), and recovery of the pre-sequencing mix in 25 □L of Elution Buffer (Oxford Nanopore Technologies). After priming the flow cell with running buffer and fuel according to the manufacturer's recommendations, an initial 6 μL aliquot of the pre-sequencing mix (at 10-20 ng/μL) was combined with 75 μL Running Buffer, 65 μL water, and 4 μL Fuel Mix and applied to the flow cell. Thereafter, at 8 hr intervals, additional pre-sequencing mix aliquots (held on ice) were combined with Running Buffer and Fuel Mix and added to the flow cell at times roughly coinciding with re-programmed pore remux (modified scripts from J. Tyson, pers. comm.) Modified remux scripts were not used for the final MinION Mk1 run (Staggered community analysis) because that run was controlled by a new version of MinKNOW.

Comparative data sets were generated for each of the four single species templates using full length ~1500 bp Sanger sequencing of a 16S amplicon. Reads from the 16S analysis were subjected to BlastN for taxonomic assignment. Data from the MinION were accessed on the MG-RAST server and annotated based on their predicted proteins and rRNA genes.

Figure 1 Result of "What's in my pot."

Rendering of real-time analysis using WIMP [20] of whole genome sequences from a synthetic mixture prepared from equal DNA quantities of four cultured microbe species (experiment "Equal Mk1") and run on the MinION™ sequencing platform. Arc angle is proportional to the number of reads assigned to the indicated species. Colors (scale at bottom of diagram) refer to the classification score threshold (for this analysis the threshold for inclusion was 0.01).

Figure 2 Principal component analysis of normalized 5-mer frequency (i.e., percentage) within each MinION™ read.

Performed for runs with equal portions of four species and with three equal and one rare species included in the DNA pool. “none”: read had no BlastN hits. “other”: read had BlastN hits but not one of the four species included in the mix.

Figure 3 Log abundance of reads assigned from staggered mixture.

DNA of 20 species mixed in various proportions (bei Resources, ATCC, HM-783D, operon counts μL^{-1} in original mixture indicated along bottom margin of bars), was pre-amplified with Φ29 polymerase prior to library preparation and sequenced with MinION™. The 2D pass reads were assigned to taxa using Kraken. Colored bars are species included in the mix whereas gray bars indicate species detected but not included in the original DNA mixture.

Figure 4 Read production using a MinION™ Mk1 device and an R7.3 flow cell.

Shows reads collected from a synthetic metagenome made with equal DNA amounts from four microbes and a library prepared using SQK–MAP006 kit. Arrows indicate approximate times when additional aliquots of library and fuel were added.

Availability and requirements

- Project name: Experimental Metagenome on MinION
- Project home page: https://github.com/gigascience/paper link will be here.
- Operating system: Unix
- Programming language: Bash and R
- Other requirements: Unix
- License: N/A

Availability of supporting data

The datasets supporting the results of this article are available in the GigaDB repository [Brown et al. 2016] and MG-RAST 4629367.3, 4629445.3, 4629369.3, 4629381.3, 4614572.3, 4685746.3, 4685745.3, 4705090.3

Competing interests

BLB, MW, MCR, and RBF are enrolled in the Oxford Nanopore MinION™ Access Programme (MAP) and received free materials for this research. KPK has no competing interests.

Authors' contributions

BLB conceived of the study, performed the DNA extraction and sequencing, directed the data analysis, and drafted the manuscript. KPK provided data analysis, bioinformatics, and annotation. MW provided bioinformatic analyses and statistical analyses. MCR participated in study design, sequence alignment, and bioinformatic analysis. RBF participated in study design, sequencing, data analysis, and manuscript preparation. All authors read and approved the final manuscript.

Acknowledgements

This work was supported by the Virginia Commonwealth University Department of Biology (BLB, MCR, RBF), by GenEco, LLC, Richmond, Virginia to BLB, and funding for MW was from the Biotechnology and Biological Sciences Research Council including Institute Strategic Programme and National Capability grants (BBSRC; BBS/E/D/20310000, BB/J004243/1, BB/M020037/1). The authors acknowledge Kensie Barker (VCU) for assistance with culturing bacteria. John Tyson (UBC) provided runtime plots and wrote the python scripts used to control the MinION device during the run. Hugh Eaves (VCU)

provided programming assistance. Sarah Highlander (Venter Inst.) provided advice on determining DNA concentration. Michael Micorescu (ONT) provided assistance with Kraken.

List of abbreviations

MAP: MinION™ Access Programme

2D: refers to sequences where both the template and the complement were completed (bidirectional) and passed the Metrichor quality threshold (Q9)

References

1. Kasianowicz JJ, Brandin E, Branton D, Deamer DW. Characterization of individual polynucleotide molecules using a membrane channel. Proc Natl Acad Sci. 1996;93:13770–73.

2. Eisenstein M. Oxford Nanopore announcement sets sequencing sector abuzz. Nat Biotechnol. 2012;30:295–6.

3. Loman NJ, Quick J, Simpson JT. A complete bacterial genome assembled de novo using only nanopore sequencing data. Nat Methods. 2015;12:733–5.

4. Goodwin S, Gurtowski J, Ethe-Sayers S, Deshpande P, Schatz M, McCombie WR. Oxford Nanopore sequencing and de novo assembly of a eukaryotic genome. Genome Res. 2015a;25:1-7.

5. Risse J, Thomson M, Blakely G, Koutsovoulos G, Blaxter M, Watson M. A single chromosome assembly of Bacteroides fragilis strain BE1 from Illumina and MinION nanopore sequencing data. GigaScience 2015;4:60.

6. Quick J, Ashton P, Calus S, Chatt C, Gossain S, et al. Rapid draft sequencing and real-time nanopore sequencing in a hospital outbreak of *Salmonella*. Genome Biol. 2015;16:114.

7. Madoui M-A, Engelen S, Cruaud C, Belser C, Bertrand L, et al. Genome assembly using nanopore-guided long and error-free DNA reads. BMC Genomics. 2015;16:327.

8. Mongan AE, Yusuf I, Wahid I, Hatta M. The evaluation on molecular techniques of reverse transcription loop-mediated isothermal amplification (RT-LAMP), reverse transcription polymerase chain reaction (RT-PCR), and their diagnostic results on MinION™ nanopore sequencer for the detection of dengue virus serotypes. Am J Microbiol Res. 2015;3:118-24.

9. Hargreaves AD, Mulley JF. Snake venom gland cDNA sequencing using the Oxford nanopore MinION portable DNA sequencer. PeerJ. 2015 Nov 24;3:e1441. doi: 10.7717/peerj.1441. eCollection 2015.

10. Bolisetty MT, Rajadinakaran G, Graveley BR. Determining exon connectivity in complex mRNAs by nanopore sequencing. Genome Biol. 2015; 16:204.

11. Cao MD, Ganesamoorthy D, Elliott A, Zhang H, Cooper M, Coin L. Streaming algorithms for identification of pathogens and antibiotic resistance potential from real-time MinION sequencing. bioRxiv 2015; doi: http://dx.doi.org/10.1101/019356.

12. Judge K, Harris SR, Reuter S, Parkhill J, Peacock SH. Early insights into the potential of the Oxford Nanopore MinION for the detection of antimicrobial resistance genes. J Antimicrob Chemother 2015; doi:10.1093/jac/dkv206.

13. Wang J, Moore NE, Deng Y-M, Eccles DA, Hall RJ. MinION nanopore sequencing of an influenza genome. Front Microbiol. 2015;6:766.

14. Kilianski A, Haas JL, Corriveau EJ, Liem AT, Willis KL, et al. Bacterial and viral identification and differentiation by amplicon sequencing on the MinION nanopore sequencer. GigaScience 2015;4:12.

15. Greninger AL, Naccache SN, Federman S, Yu G, Mbala P, et al. Rapid metagenomic identification of viral pathogens in clinical samples by real-time nanopore sequencing analysis. Genom Med. 2015;7:99.

16. Brown BL, Keegan KP, Watson M, Rivera MC, Franklin RB. Whole genome data from synthetic metagenomes. GigaScience Database. 2016. http://xxxxxx.

17. Watson M, Thomson M, Risse J, Talbot R, Santoyo-Lopez J, et al. poRe: an R package for the visualization and analysis of nanopore sequencing data. Bioinformatics. 2015;31:114-5.

18. Loman NJ, Quinlan AR. Poretools: a toolkit for analyzing nanopore sequence data. Bioinformatics. 2014; 30:3399–3401.

19. Leggett RM, Heavens D, Caccamo M, et al.: NanoOK: Multi-reference alignment analysis of nanopore sequencing data, quality, and error profiles. Bioinformatics. 2015;32:142-4.

20. Juul S, Izquierdo F, Hurst A, Dai X, Wright A, Kulesha E, Pettett R, Turner D. What's in my pot? Real-time species identification on the MinION™. bioRxiv. 2015; doi: http://dx.doi.org/10.1101/030742.

21. Altschul SF, Madden TL, Schaffer AA, Zhang J, Zhang Z, Miller W, Lipman DJ. Gapped BLAST and PSI-BLAST: a new generation of protein database search programs. Nucl Acids Res. 1997;25:3389.

22. Wood DE, Salzberg SL. Kraken: ultrafast metagenomic sequence classification using exact alignments. Genome Biol. 2014;15:R46.

23. Meyer F, Paarmann D, D'Souza M, Olson R, Glass EM, et al. The metagenomics RAST server – a public resource for the automatic phylogenetic and functional analysis of metagenomes. BMC Bioinfo. 2008;9:386.

24. R Core Team. R: A language and environment for statistical computing. R Foundation for Statistical Computing, Vienna, Austria. 2015;https://www.R-project.org/.

25. Park H-D, Sasaki Y, Maruyama T, Yanagisawa E, Hiraishi A, Kato K. Degradation of the cyanobacterial hepatotoxin microcystin by a new bacterium isolated from a hypertrophic lake. Environ Toxicol 2001;16:337-43.

26. Thomas T, Gilbert J, Meyer F. Metagenomics – a guide from sampling to data analysis. Microb Inform Experim. 2012;2:3.

27. Jones MB, Highlander SK, Anderson EL, Li W, Dayrit M, et al. Library preparation methodology can influence genomic and functional predictions in human microbiome research. Proc Nat Acad Sci. 2015;45:14024-9.

28. Wommack KE, Bhavsar J, Ravel J. Metagenomics: Read Length Matters. Appl Environ Microbiol. 2008;74:1453.

29. Brown BL, LePrell RV, Franklin RB, Rivera MC, Cabral FC, et al. Metagenomic analysis of planktonic microbial consortia from a non-tidal urban-impacted segment of James River. Stand Genomic Sci. 2015;10:65.

30. Magasin JD, Gerloff DL. Pooled assembly of marine metagenomic datasets: enriching annotation through chimerism. Bioinformatics. 2015;31:311-317.

31. Freitas TAK, Li P-E, Scholz MB, Chain PSG. Accurate read-based metagenome characterization using a hierarchical suite of unique signatures. Nucl Acids Res. 2015; doi: 10.1093/nar/gkv180.

32. Li C, Chng KR, Boey EJH, Ng AHQ, Wilm A, Nagarajan N. INC-Seq: accurate single molecule reads using nanopore sequencing. GigaScience. 2016;5:34

33. Benítez-Páez A, Portune KJ, Sanz Y. Species-level resolution of 16S rRNA gene amplicons sequenced through the MinION™ portable nanopore sequencer. GigaScience. 2016;5:4.

34. Goodwin S, Gurtowski J, Ethe-Sayers S, Deshpande P, Schatz M, McCombie WR. Oxford Nanopore sequencing, hybrid error correction, and *de novo* assembly of a eukaryotic genome. Genome Res. 2015b;25:1750-6.

35. Karlsson E, Lärkeryd A, Sjödin A, Forsman M, Stenberg P. Scaffolding of a bacterial genome using MinION nanopore sequencing. Scientif Rep 2015;5:11996.

36. Stephens ZD, Lee SY, Faghri F, Campbell RH, Zhai C, et al. Big data: astronomical or genomical? PLoS Biol. 2015;13:e1002195. doi:10.1371/journal.pbio.1002195.

37. Ip CLC, Loose M, Tyson JR, Cesare M, Brown BL, et al. MinION Analysis and Reference Consortium: Phase 1 data release and analysis. F1000Research 2015;4:1075.

Figure 1

Click here to download Figure
Figure1BrownetalMinIONMetagenome.docx

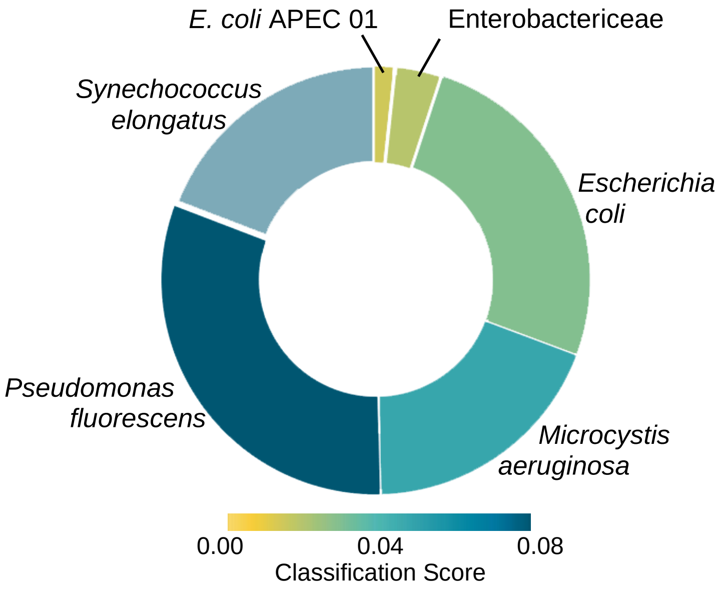

Figure 2

Click here to download Figure
Figure2BrownetalMinIONMetagenome.docx

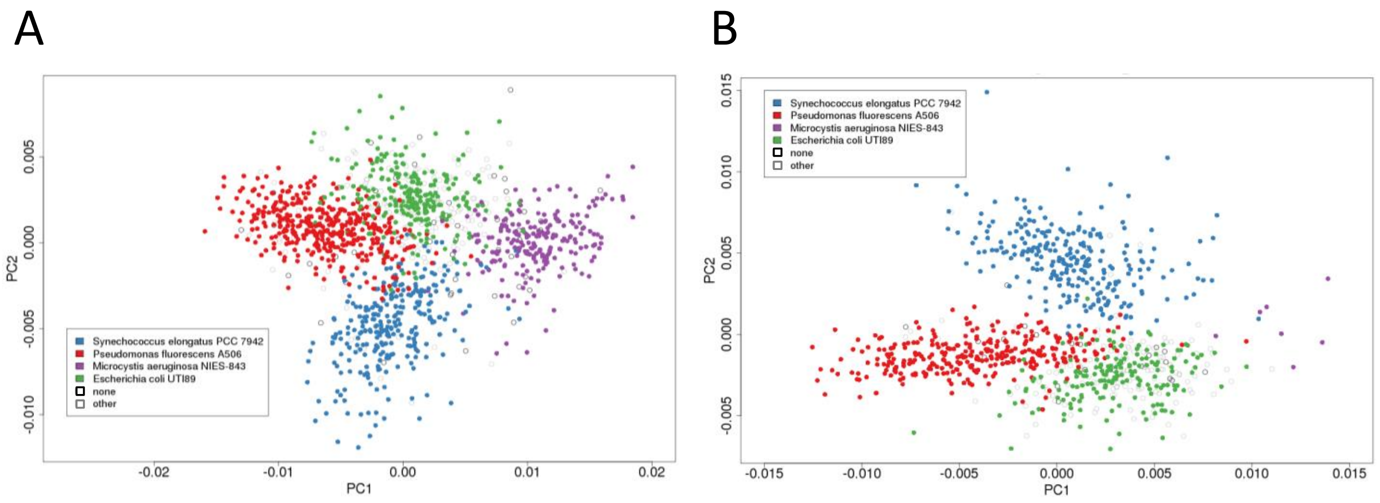

Figure 3

Click here to download Figure
Figure3BrownetalMinIONMetagenome.docx

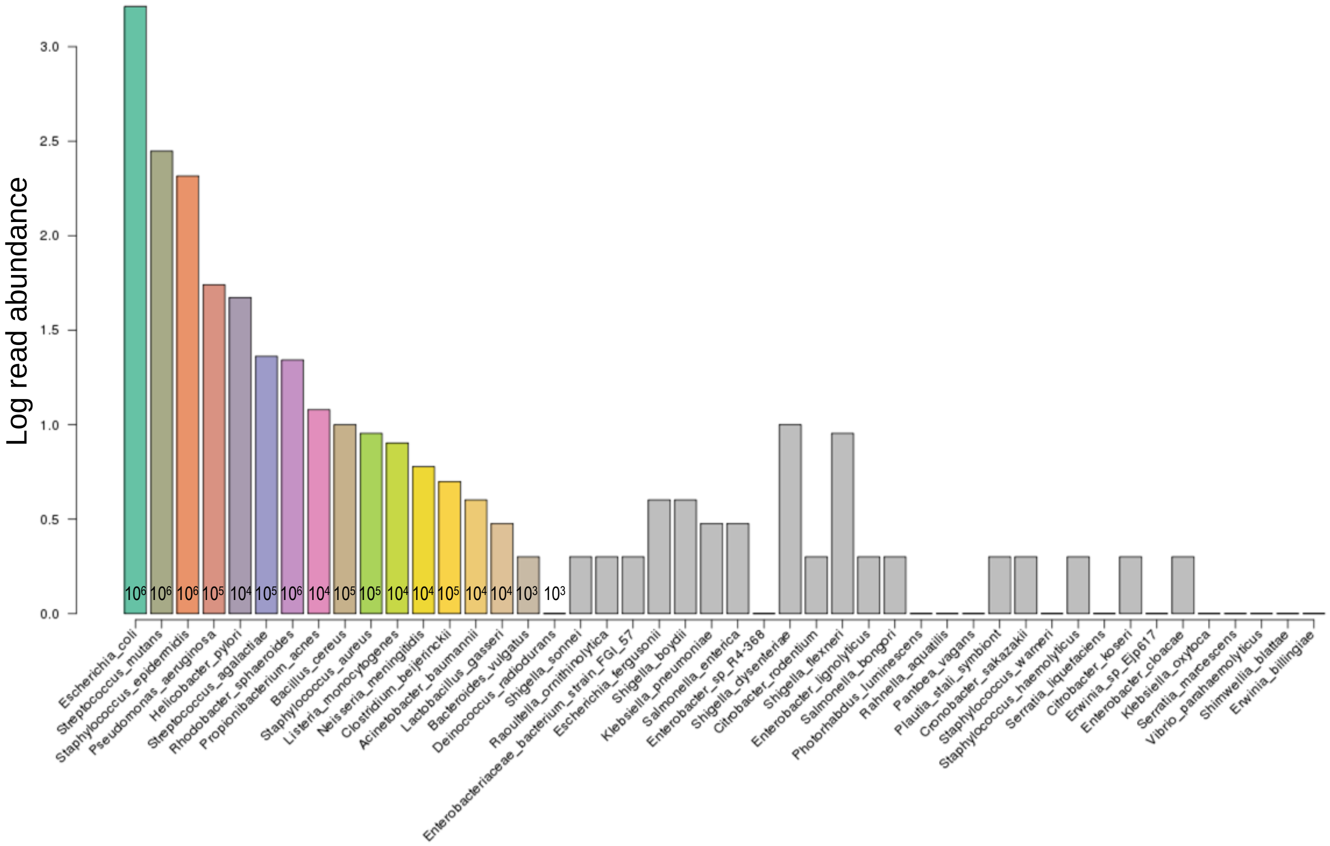

Figure 4

Click here to download Figure
Figure4BrownetalMinIONMetagenome.docx

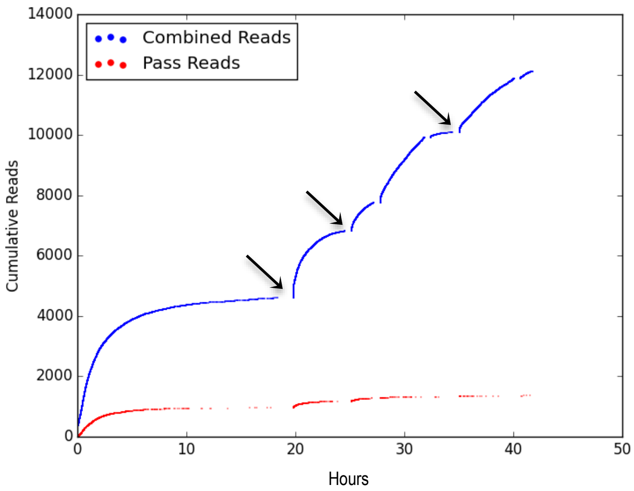

Personal Cover
Click here to download Personal Cover CoverLetterBrownetalMinionMetagenome.pdf

VIRGINIA COMMONWEALTH UNIVERSITY

VCU

Virginia Commonwealth University
College of Humanities and Sciences
Department of Biology
Ecological Genetics Laboratory
1000 West Cary Street
P.O. Box 842012
Richmond, Virginia 23284-2012
804-828-3265 voice
804-828-0503 fax
800-828-1120 TDD

16 August 2016

We respectfully submit the attached manuscript entitled, "MinION™ nanopore sequencing of environmental metagenomes: a synthetic approach," for consideration for publication in GigaScience.

We have selected GigaScience as the venue to share our results with the world for two primary reasons. First, a numbe of other MinION papers are located in this journal so our work will likely be seen by others who are considering use of this sequencing platform. Second, because a number of other metagenomic studies are published in this journal, we believe that our work will be noted by researchers who are

Our manuscript is worthy of publication in GigaScience because it is well-conceived, considers in depth the consequences of long read sequencing of both simple and complex mixtures, and provides clear guidance for selecting analytical tools for long read sequence data analysis.

We take no exception to any issues relating to journal policies.

We have declared in the manuscripts our potential competing interests as follows: the contact author, Bonnie Brown, along with two other VCU authors (Maria Rivera and Rima Franklin) are members of the VCU MinION Access Programme. Mick Watson also is a member of this program. Kevin Keegan is not and has no other identifiable competing interests.

All authors have contributed to writing and have approved the manuscript for submission.

The content of this manuscript has not been published, nor has it been submitted for publication, elsewhere.

Sincerely,

Bonnie L. Brown, Ph.D.
Professor and Associate Chair of Biology
Director, VCU Ecological Genetics Laboratory
