# Supplementary material for: MinION™ nanopore sequencing of environmental metagenomes: a synthetic approach
Source: Gigascience. 2017 Feb 24;6(3):1–10. doi: 10.1093/gigascience/gix007 (PMC5467020; doi:10.1093/gigascience/gix007)

Manuscript
Click here to download Manuscript GigaScienceSynthMetagenomAccepMinorRev.docx

MinION™ nanopore sequencing of environmental metagenomes: a synthetic approach

Bonnie L. Brown, Virginia Commonwealth University, Department of Biology, 1000 W Cary Street, Richmond, VA 23284, USA, blbrown@vcu.edu

Mick Watson, The Roslin Institute, University of Edinburgh, Division of Genetics and Genomics, Easter Bush, Midlothian, EH25 9RG, UK, mick.watson@roslin.ed.ac.uk

Samuel S. Minot, One Codex, 165 11th St, San Francisco, CA 94103, USA, sam@onecodex.com

Maria C. Rivera, Virginia Commonwealth University, Department of Biology, 1000 W Cary Street, Richmond, Virginia 23284, USA, mcrivera@vcu.edu

Rima B. Franklin, Virginia Commonwealth University, Department of Biology, 1000 W Cary Street, Richmond, Virginia 23284, USA, rbfranklin@vcu.edu

Corresponding author: Bonnie L. Brown, blbrown@vcu.edu

Abstract

Background: Environmental metagenomic analysis is typically accomplished by assigning taxonomy and/or function from whole genome sequencing (WGS) or 16S amplicon sequences. Both of these approaches are limited, however, by read length, among other technical and biological factors. A nanopore-based sequencing platform, MinION™, produces reads that are $\geq 1\times10^4$ bp in length, potentially providing for more precise assignment, thereby alleviating some of the limitations inherent in determining metagenome composition from short reads. We tested the ability of sequence data produced by MinION (R7.3 flow cells) to correctly assign taxonomy in single bacterial species runs and in three types of low complexity synthetic communities: a mixture of DNA using equal mass from four species, a community with one relatively rare (1%) and three abundant (33% each) components, and a mixture of genomic DNA from 20 bacterial strains of staggered representation. Taxonomic composition of the low-complexity communities

was assessed by analyzing the MinION sequence data with three different bioinformatic approaches : Kraken, MG-RAST, and One Codex.

Results: Long read sequences generated from libraries prepared from single strains using the version 5 kit and chemistry, run on the original MinION device, yielded as few as 224 to as many as 3,497 bidirectional high-quality (2D) reads with an average overall study length of 6,000 bp. For the single-strain analyses, assignment of reads to the correct genus by different methods ranged from 53.1% to 99.5%, assignment to the correct species ranged from 23.9% to 99.5%, and the majority of mis-assigned reads were to closely related organisms. A synthetic metagenome sequenced with the same setup yielded 714 high quality 2D reads of approximately 5,500 bp that were up to 98% correctly assigned to the species level. Synthetic metagenome ~~from~~ MinION libraries generated using version 6 kit and chemistry yielded 899-3,497 2D reads with lengths averaging 5,700 bp with up to 98% assignment accuracy at the species-level. The observed community proportions for “equal” and “rare” synthetic libraries were close to the known proportions, deviating from 0.1 – 10% across all tests. For a 20-species mock community with staggered contributions, a sequencing run detected all but 3 species (each included at <0.05% of DNA in the total mixture); 91% of reads were assigned to the correct species, 93% of reads were assigned to the correct genus, and >99% of reads were assigned to the correct family.

Conclusions: At the current level of output and sequence quality (just under 4×10^3 2D reads for a synthetic metagenome), MinION sequencing followed by Kraken or One Codex analysis has the potential to provide rapid and accurate metagenomic analysis where the consortium is comprised of a limited number of taxa. Important considerations noted in this study included: high sensitivity of the MinION platform to the quality of input DNA, high variability of sequencing results across libraries and flow cells, and relatively small numbers of 2D reads per analysis limit. Together, these limited detection of very rare components of the microbial consortia, and would likely limit the utility of MinION for the sequencing of high-complexity metagenomic

communities where thousands of taxa are expected. Furthermore, the limitations of the currently available data analysis tools suggest there is considerable room for improvement in the analytical approaches for the characterization of microbial communities using long reads. Nevertheless, the fact that the accurate taxonomic assignment of high quality reads generated by MinION is approaching 99.5% and, in most cases, the inferred community structure mirrors the known proportions of a synthetic mixture, warrants further exploration of practical application to environmental metagenomics as the platform continues to develop and improve. With further improvement in sequence throughput and error rate reduction, this platform shows great promise for precise real-time analysis of the composition and structure of more complex microbial communities.

Keywords

MinION™, Oxford Nanopore Technologies, metagenome, whole-genome sequencing, long-read sequencing

Background

Environmental metagenomics, employing whole genome sequence analysis to identify ecologically and epidemiologically important components of sediments, soils, waters, and surfaces, is rapidly evolving through advances in both hardware and software [1]. Knowledge of the consortia that inhabit these ecosystems allows for better understanding of the organisms and their ecological roles, provides for the development of effective strategies to mitigate ecosystem damage, and facilitates evaluation of the responses of species to environmental change. One common approach in environmental metagenomics involves sequencing and subsequent annotation of whole genome nucleic acid fragments (WGS) extracted directly from environmental samples to discover major microbial members of the ecosystem; if sequenced deeply enough, rare species can be detected [2]. For well-studied members of the microbial community, such metagenomic data also can be used to characterize the functional potential of complex communities.

One technique for characterizing environmental metagenomes is to use short-read high-throughput sequencing followed by mapping the reads to reference genomes. Profiling the taxonomic composition of the community also can be accomplished by the analysis of the distribution of k-mers (e.g., using Kraken or One Codex). Although these methodologies are very powerful due to the depth of sequencing, the capacity to resolve the taxonomy of the community to the species level is limited by read length. One approach to overcome this limitation is to assemble short reads into contigs prior to analysis and annotation. If assembled correctly, the longer sequence lengths of the contigs have a greater chance of accurately identifying the members of the community; however, due to the mixed nature of the samples, such assembly approaches are challenged by many artifacts including chimeric contigs that inappropriately combine sequence reads from multiple species. The high information content of very long reads such as those provided by MinION™ (Oxford Nanopore Technologies, Inc., Oxford, UK) has the potential to overcome some of the limitations of short reads by allowing for longer alignments that potentially can contribute to higher taxonomic specificity, functional characterization, and resolution. Although conceived almost two decades ago [3], nanopore-based whole-molecule sequencing has only recently become available to MinION™ Access Programme (MAP) participants for exploration and practical application [4]. Data generated by early access MinION™ flow cells have been assessed for whole genome sequencing [5, 6, 7, 8, 9], gene expression and transcriptome studies [10, 11, 12], clinical applications such as inferring antibiotic resistance of bacterial strains and the detection of influenza and Ebola virus [13, 14, 15], bacterial and viral serotyping [16], and clinical metagenomes of viral pathogens [17]. Efforts to use this technology to study diverse environmental communities have been limited [18] and there has not been, to our knowledge, any cross-validation of the results or any systematic assessment to determine the best data analysis strategies for nanopore-based environmental metagenomics. To investigate the potential of this platform for broader applications, we performed a set of experiments to quantify the ability of MinION™ long-read sequence data to accurately characterize the taxonomic composition and structure of metagenomes by assessing its performance in the characterization of low complexity synthetic metagenomes.

Data description

The raw MinION data [19] collected during sequencing by MinKNOW software (versions 0.49.2.9 through 0.51.3.40 b201605171140) were immediately uploaded as FAST5 packets to Metrichor Agent (r7.3 2D basecalling, ver rx-2.22-44717-dg-1.6.1-ch-1.6.3; Mk1 2D base-calling, ver WIMP Bacteria k24 for SQK-MAP006), after which base-called data [19] were returned to the host computer, also in the form of FAST5 files. The programs poRe [20], Poretools [21], and NanoOK [22] were used to extract and characterize the numbers of reads and channels, after which only the 2D reads were stored in FASTQ and FASTA files for downstream analyses. The base-called data sets were scrutinized by methods commonly employed in metagenome analysis of short reads including MG-RAST [23], which assigns taxonomy based on predicted proteins and rRNA genes. The data sets also were analyzed by tools that have been shown to work for long-read data including: (1) WIMP [24], which assigns taxonomy by comparing read sequences against a database of bacteria, (2) Kraken [25], which uses exact alignments of *k-mers* and indexes more than 5000 genomes and plasmids, (3) One Codex [26], which uses exact *k*-mer alignment to classify sequences against a reference database of ~40,000 complete microbial genomes (including bacteria, viruses, fungi, protists, and archaea), and (4) by principal components analysis (PCA) based on the frequency of 5-mers in each read followed by annotation of reads with the top BlastN [27] hit (carried out in R [28]). Specific parameters are described in Methods.

Results

MinION™ WGS libraries were generated from 1 μg of fresh DNA isolates (see Methods) of separate cultures of two Proteobacteria, *Escherichia coli* and *Pseudomonas fluorescens*, and two Cyanobacteria, *Microcystis aeruginosa*, and *Synechococcus elongatus*, and from two different DNA mixtures of these four species. One mixture combined an equal mass of genomic DNA (gDNA) from each of the four species. The other mixture was created by combining 33% mass of gDNA from each of three species and only 1% of gDNA mass from the other species. The preparation of these libraries

yielded sufficient Pre-sequencing Mix for multiple loads of each flow cell. An additional library was derived from a commercially prepared 20-species mock community. Because only 100 ng of material was provided by the supplier, genome pre-amplification using Φ29 polymerase was required to generate sufficient mass of DNA to create the sequencing library (see Methods).

To assess the purity of the cultures used in this study, we used the Sanger method to sequence full-length (~1500 bp) 16S amplicons from each (Table 1). Inspection of those data revealed varying degrees of genomic uniqueness at the species level. For the strain of *M. aeruginosa* used in this study, the top 16S hit had a low sequence identity to any reference sequence in the database (90%). In contrast, the input strain of *S. elongatus* was 99% identical to two different species of *Synechococcus* (*S. elongatus* and *S. UTEX 2973).* In addition, whole-genome alignment indicated that the input strain of *P. fluorescens* was highly similar to multiple species of *Pseudomonas*. However, all of the input organisms were distinct at the genus level, thus that taxonomic level was used for downstream analysis of the single-species and 'Equal' and 'Rare' synthetic samples.

MinION sequencing of the single-species libraries generated up to $31{\times}10^3$ reads ($0.2–1.1{\times}10^3$ 2D reads that passed the quality filter) ranging from as short as 5 bp to as long as $267{\times}10^3$ bp (data include both 2D pass and fail reads), and the resulting average length of single-species read subjected to downstream analysis was $6{\times}10^3$ bp. Using MG-RAST, Kraken, and One Codex, up to 99.5% of the high quality 2D reads obtained from the sequencing of the single-species libraries of *E. coli*, *P. fluorescens*, *S. elongatus*, and *M. aeruginosa* were taxonomically assigned to the corresponding input taxa (Table 3). The least accurate assignments were for *M. aeruginosa*, where at best 58% of 2D reads were correctly assigned to the level of species, although more than half of the mis-assigned reads were to closely related cyanobacteria genera and other prokaryotes known to break down microcystin [29] (data not shown). All three methods of analysis assigned sequence reads of the *P. fluorescens* single-species library to *Stenotrophomonas*. Over all of these analyses, MG-RAST generally showed the lowest rate of correct taxonomic assignment and, although One Codex and Kraken provided similar results, Kraken showed a lower rate of correct assignment for *M. aeruginosa* (85%) compared to One Codex (95%).

In the second round of validation, using three synthetic communities containing mixtures of the previously described species, 6–12×10^3 reads (0.7–1.3×10^3 2D reads) were generated per run, ranging in length from 0.6–56.8×10^3 bp (Table 2). For the two communities comprised of equal DNA contribution from four bacteria (25% each species), WGS proportions accurately aligned with the known proportions 87–99% of the time when analyzed using Kraken or One Codex and 65–85% using MG-RAST (Table 3). Specifically, taxonomic assignment of reads obtained from the sequencing of the equal mixture of four species (25% of each) using version 5 chemistry and run on an original MinION device identified the following taxa: 27% *E. coli*, 16% *M. aeruginosa*, 30% *P. fluorescens*, 21% *S. elongatus*, 3% Enterobacteriaceae, and 3% misclassified. In a subsequent test (version 6 chemistry), classification results for the equal mixture were: 26% *E. coli*, 18% *M. aeruginosa*, 30% *P. fluorescens*, 22% *S. elongatus*, and 3% Enterobacteriaceae, and 1% misclassified (Figure 1). For the community with three common (33% of each) and one rare (1%) representative, classifications were: 33% *E. coli*, 34% *P. fluorescens*, 29% *S. elongatus*, 1% *M. aeurginosa*, 2% misclassified (a third of those latter category of reads were assigned to *Shigella*). For both the "Equal" and "Rare" community data sets, the 5-mer frequency profiles were computed and visualized using the top BlastN hit for each full read, revealing that 5-mer profiles for these long-read sequences were shared within species. This was reflected in the 5-mer frequency analysis which revealed distinct per-species clusters in the PCA plots (Figure 2).

In the final round of testing, the mock microbial community with 20 species included in "staggered" proportions (i.e., 1,000 to 1,000,000 16S rRNA operon copies per organism per μL of material supplied by BEI Resources, Catalog # HM-783D) yielded 14.7×10^3 reads (3.5×10^3 2D reads) ranging in length from 0.5–20.9×10^3 bp, sufficient to detect all of the high and moderate abundance species, but the sequencing run failed to detect 3 of 5 species that were included at very low mass (0.6–1.0 pg/μL of material supplied; Table 4). For that run, misclassifications accounted for only 0.2% of read assignments but greatly overrepresented in the results for this run were reads assigned to *E. coli* (included as 20% of DNA but observed as 46-52% of read assignments), whereas greatly underrepresented in the results were reads assigned to *R. sphaeroides*, which was putatively included

as 41% of DNA mass but accounted for only 1% of read assignments (Figure 3). Although 75% of the read assignments made by WIMP were to genera known to comprise the mock community, 93% of the read assignments made by One Codex matched the correct genera.

Table 1 Identity of single-species used in this study as determined by Sanger sequencing of 16S rDNA amplicons from different DNA preparations of each species.

Culture [a]	Final sequence Length (bp)	Sequence matches in BlastN	
		%	Organism
Escherichia coli	1440–1696 [a]	98	*E. coli* numerous strains
Microcystis aeruginosa	1418	90	*M. aeruginosa* NIES-843 and NIEHS-2549, and *M. panniformis* FACHB-1757
Pseudomonas fluorescens	1478–1570	96	*P. fluorescens* A506 and LBUM223
Synechococcus elongatus	1431–1719	99	*S. elongatus* PCC 7942, PCC 6301, UTEX 2973

[a] Multiple DNA preparations from bacterial cultures were used during the progress of the study, and each was tested, yielding for each strain slightly different final 16S sequence lengths, but the same BLAST matches.

Table 2 Details of MinION™ whole genome sequencing output for single-species and synthetic mixtures. Sequencing experiments used the MinION device and new R7.3 flow cells. Libraries were prepared with kit SQK–MAP005 as indicated by (5) and SQK-MAP006 chemistry, indicated by (6). Columns relating to "2D" indicate bi-directional reads with quality above Q9.

Experiment (chemistry)	Pores with reads	Run time (hr) [a]	Total bp (Mbp)	Total reads	Number of 2D pass reads	Mean 2D read length (bp)	MG-RAST accession	ENA accession
Single species								
E. coli (5)	430	42	83.6	26590	1112	5274	4629367.3	ERR1713483
P. fluorescens (5)	453	48	119.4	25228	777	7784	4629445.3	ERR1713487

M. aeruginosa (5)	377	18	40.8	22760	569	5676	4629369.3	ERR1713486
S. elongatus (5)	367	23	18.3	6163	224	5101	4629381.3	ERR1713489
Mixtures								
Equal (5)	129	24	26.5	10592	714	5527	4614572.3	ERR1713484
Equal (6)	437	44	77.1	12174	1358	5202	4685746.3	ERR1713485
Rare (6)	449	18	39.0	6728	899	6194	4685745.3	ERR1713488
Staggered (6)	300	33	39.0	14711	3497	2612	4705090.3	ERR1713490

[a] Runs were set to either 24 or 48 hours and were allowed to continue until either sufficient sequence data were collected or until the 2D pass rate was greatly reduced.

Table 3 Taxonomic assignment accuracy of metagenomic reads across three analysis methods.

	Accuracy of assignment to known genus (%)		
**Experiment**	**MG-RAST**	**Kraken**	**One Codex**
Single species			
E. coli (5)	74.4 [a]	99.5	98.7
P. fluorescens (5)	84.9 [b]	84.6 [b]	84.2 [b]
M. aeruginosa (5)	53.1	85.8	95.1
S. elongatus (5)	87.9	98.1	97.6
Mixtures			
Equal (5)	65.0 [b]	97.6	87.4 [c]
Equal (6)	85.9	98.0	98.7
Rare (6)	92.9	99.1	98.7

[a] 15% of reads assigned to *Shigella*

[b] 7-15% of reads assigned to *Stenotrophomonas*

[c] 7% of reads assigned to *Stenotrophomonas*

Accuracy was calculated as the proportion of reads assigned to the known input organism at the genus level out of the total number reads given any assignment at that rank.

Table 4 Known composition of 20-species mock staggered community compared with analysis results for WIMP and One Codex. "nd": not detected.

Organism	Operon count/µL [a]	Quantity pg/µL [b]	% DNA in template [c]	WIMP % species	WIMP % genus	One Codex % species	One Codex % genus

Acinetobacter baumannii	10,000	8.2	0.24	0.14	0.14	0.29	0.29
Actinomyces odontolyticus	1,000	1	0.03	nd	nd	nd	nd
Bacillus cereus	100,000	45	1.33	0.53	0.53	0.66	0.75
Bacteroides vulgatus	1,000	0.8	0.02	0.1	0.1	0.07	0.12
Clostridium beijerinckii	100,000	44	1.30	0.19	0.19	0.29	0.35
Deinococcus radiodurans	1,000	1	0.03	0.05	0.05	0.07	0.06
Enterococcus faecalis	1,000	0.7	0.02	nd	nd	nd	nd
Escherichia coli	1,000,000	680	20.04	45.61	45.66	52.15	52.52
Helicobacter pylori	10,000	8.6	0.25	1.68	1.68	3.43	2.72
Lactobacillus gasseri	10,000	3.2	0.09	0.14	0.14	0.22	0.23
Listeria monocytogenes	10,000	5	0.15	0.38	0.38	0.58	0.52
Neisseria meningitidis	10,000	5.8	0.17	0.24	0.24	0.44	0.41
Propionibacterium acnes	10,000	8.8	0.26	0.48	0.48	0.07	0.64
Pseudomonas aeruginosa	100,000	160	4.71	1.25	1.25	3.07	3.18
Rhodobacter sphaeroides	1,000,000	1,400	41.25	1.01	1.01	1.46	1.27
Staphylococcus aureus	100,000	59	1.74	0.38	3.88	1.31	12.74
Staphylococcus epidermidis	1,000,000	510	15.03	7.67	7.72	6.65	
Streptococcus agalactiae	100,000	32	0.94	0.96	1.01	0.95	
Streptococcus mutans	1,000,000	420	12.38	10.17	10.17	19.50	16.97
Streptococcus pneumoniae	1,000	0.6	0.02	nd	nd	nd	
Other		0	0	29.02 [d]	25.37 [e]	8.77 [f]	7.24 [g]
Correct assignments				70.98	74.63	91.23	92.76

[a] Theoretical copy number provided by BEI Resources certificate of analysis
[b] gDNA content provided by BEI Resources certificate of analysis
[c] Proportion of individual species within the mock community.
[d] Of these, 12.7% were correctly assigned to genus, 86.4% were Enterobacteriaceae, and only 0.7% were misclassifications.
[e] Of these, 86.4% were Enterobacteriaceae and only 0.7% were misclassified.
[f] Of these, 56.8% were *Shigella*.
[g] Of these, 63.3% were species of *Escherichia* and *Shigella*.

Discussion

Sequencing of whole genome libraries can enhance environmental metagenomic analysis by providing more precise identification of the composition and structure of the community than is

possible by amplicon sequencing of marker genes (e.g., 16S) [2, 30]. Typical environmental samples contain tens of thousands to millions of organisms, yet the resulting metagenomes almost certainly underrepresent this diversity and, often due to short-read strategy, the resulting data sets can be confidently assigned only to higher taxonomic levels [31, 32]. One strategy to improve the accuracy of taxonomic assignment is to carefully assemble metagenomic data, which despite the potential for chimeric contig formation has been shown to greatly enhance species call correctness [33]. However, even with enhanced sequencing and bioinformatic strategies, many public database accessions contain sequences that are not innate to the species that was analyzed; these include symbionts, parasites, pathogens, and sequencing linkers/primers/adapters (unbeknownst to those who have accessed the data) that can lead to false discovery rates [34]. Contaminated and mis-annotated reference sequences can affect environmental metagenome analyses that are derived from short reads to a greater extent than would be expected from analyses based on long reads. Long reads can circumvent these issues [31, 35, 36], so long as much of the genome for each component organism is represented in the sequencing library and there are few errors in the sequences and the reference database. The results reported here allow us to consider the potential utility of MinION long read sequencing and subsequent bioinformatic analysis for shotgun environmental metagenomics.

The primary challenge of microbial metagenomic sequence analysis using long reads is the comparison of input sequences against a large reference database of whole genomes from bacteria, viruses, fungi, etc. Although a number of algorithms have been developed for alignment of long, error-prone reads [37, 38], those sensitive algorithms are not optimized for the challenge of comparison against the large and ever-expanding universe of microbial genomes. The bioinformatic methods used in this analysis, MG-RAST, Kraken, One Codex, and WIMP, each compare the input reads against their own more concise reference databases, providing an assignment for the most likely origin of each individual sequence.

We found that for low complexity synthetic communities, long reads generated by MinION provided sufficiently precise sequence data to assign organisms represented at or above 1%. In fact, two out of five species included at <0.05% in a mock community (and 9 out of 9 species included at 0.05-1.00%) were detected. Furthermore, for un-amplified whole genome preparations, read

assignments were observed to be within about 10% of their proportional occurrence in the metagenome. Ultimately, we saw that although the reads were longer, because the sequence coverage was not as deep, the improvement in specificity of assignment was offset by a reduction in the sensitivity, and some of the genomes present at low concentration were not detected.

By comparing the output of multiple analysis methods, we were able to gain insight into the performance of various bioinformatic approaches for analyzing error-prone MinION reads. Overall, MG-RAST provided the lowest level of accuracy and detected multiple organisms that were not a part of the known input set. This is not surprising given that MG-RAST is optimized for analyzing short-read, low-error data. Kraken and One Codex performed similarly for the single-species samples except in the case of *M. aeruginosa*, in which case One Codex correctly identified this taxon at a higher rate than Kraken (95% *vs*. 85%). For the equal mixture with the version 5 chemistry, Kraken showed a higher rate of correct assignment than One Codex (97.6% vs 87.4%), although the two methods were generally comparable (actually One Codex was slightly more accurate) for the equal mixture when using version 6 of the MinION chemistry. An unexpected finding of this study was the detection by all three methods of *Stenotrophomonas* in the *P. fluorescens* single-species sample. Interestingly, *Stenotrophomonas* was classified as *Pseudomonas* when it was first discovered, based on similar metabolic capabilities, and was later moved to its own genus based on molecular data [39]. Our 16S sequences derived from laboratory cultures used in this study did not identify *Stenotrophomonas*, suggesting that its identification in the mixed metagenomes is not a result due to a contaminant but rather, an artifact caused by assigning taxonomy to reads with multiple sequencing errors. Also contributing to its identification is the fact that both *Pseudomonas* and *Stenotrophomonas* share functional phenotypic characteristics, indicating they may share homologous genes coding for those characteristics. The sharing of homologous genes, similar GC contents (both species genomes have 66% GC), and the higher error rate are the most likely factors responsible for the assignment of *Pseudomonas* sequence reads to *Stenotrophomonas*.

The fact that the estimated proportions of community members in synthetic mixtures were not precise despite careful DNA quantitation could indicate differences across library preparation (all libraries were prepared by BLB), reagent kits, flow cells, MinKNOW control scripts, the quality of

DNAs used to create the synthetic metagenomes, and the methods used for quantification (Qubit for the home-grown mixtures and UV spectrophotometry for the 20-species mixture). Because DNA quality is of paramount importance for MinION sequencing, PreCR (used in the version 5 protocol) or FFPE Repair Mix (used in the version 6 protocol) was included in the preparation of all libraries. The potential for profound effects related to library preparation recently was examined by Jones and collaborators [30], leading to the recommendation that studies of complex metagenomes should be based on PCR-free approaches. The current data indicate that the MinION lends itself well to a PCR-free approach but its utility for the analysis of complex metagenomes is presently limited by the small number of reads that pass the quality filtering process. The current study also provides data for considering alternatives to PCR for amplification, in this case GenomiPhi™, which was used to generate sufficient DNA for one library in the current study ("Staggered"). This method is optimized for linear DNA and was intended to generate unbiased copies of the 20-species genomes. Nevertheless, the Φ29 pre-amplification step is one possible reason for the overrepresentation of *E. coli* and underrepresentation of *R. sphaeroides* in the sequencing of the 20 species mock community. Also, a consequence of Φ29 pre-amplification combined with putative differences in DNA quality, chimeric amplicons (known to occur with Φ29 amplification of microbial communities [40]) could have been formed predominantly from higher quality *E. coli* DNA re-priming itself [41] leading to overrepresentation of the *E. coli* component. Notably, a novel low input DNA approach recently reported [42] could enhance MinION analyses of samples with low DNA yields. Although the pre-amplification step is the most likely culprit, an additional effect that could contribute to incongruence of known and estimated proportions in the 20 species mock community is that organisms for which there are many accessions in the public databases provide for more precise classification (e.g., NCBI has more than 6×10^5 *E. coli* complete genome accessions) and that *vice versa*, organisms with relatively few accessions (e.g., NCBI has only 116 *R. sphaeroides* complete genome accessions) result in less precise classification.

Despite the rather small number of 2D reads that were observed to pass the quality filter across all MinION runs, there was a strong biological signal in the data (Figure 2). Thus, as investigators have

found MinION useful for single genome introspection [6, 9, 15], 16S and other amplicon resolution [16, 43], cDNA sequencing [11], and assembly [5, 44, 45], our findings imply that this platform has immediate utility for analysis of very simple mixtures (e.g., serum testing for pathogens). Over the 18-month period of MinION use for this set of experiments, 2D pass rates increased from 2% to 24%. Because the rate of improvement is concurrent with Moore's Law [46], we speculate that future improvements will make the MinION platform very useful in the analysis of complex metagenomic samples in the near future. The cloud-based WIMP base-calling and taxon prediction program associated with the device provides a method of real-time analysis of metagenomic data. However, because we had no control over the comparative database, the cloud implementation of WIMP was far less flexible for environmental metagenomic analysis than Kraken or One Codex, and we note that use of an incomplete database can lead to false positives and negatives. By the time of submission of this study, the R7.3 flow cells and sequencing chemistry were no longer available. Subsequent versions of the platform have shown dramatically lower error and higher throughput. This study nevertheless provides a baseline for considering nanopore metagenomics and provides an impetus for further development of MinION output and data analysis, specifically with regard to evaluation of the informative value of 1D reads, scrutiny of reference data, alternative alignment algorithms, and more sophisticated k-mer analyses. As the quality rate for this platform improves, the potential will increase for MinION to accurately resolve the diversity and composition of many of the taxa in an environmental metagenome.

Methods

To set a baseline of expectations for MinION metagenomic analysis, we performed single-species sequencing runs with four organisms. Cell cultures at log phase were harvested by spinning 15 mL culture tubes at 3,000 × g for 30 min, and DNA was isolated using the PowerSoil DNA kit (MoBio, Carlsbad, CA, USA) according to the manufacturer's instructions. Nucleic acid quality and quantity were checked via Nanodrop 2000 and Qubit, whereafter 1 μg of DNA was used to prepare sequencing libraries. For the first two mixtures, equal portions of DNAs from all four organisms (250 ng each)

were used ("equal") and, for the third mixture ("rare"), equivalent amounts of three of the species were used (330 ng each) and *M. aeruginosa* was included as only 1% of the mixture (10 ng). An additional preparation of a mock community containing DNA of 20 bacterial species in staggered amounts was obtained from a commercial source (Catalog # HM-783D, BEI Resources, ATCC, Manassas, VA, USA). This mock community preparation was chosen because it previously has been used to test the ability of the R7.3 version MinION to study microbial diversity via 16S amplicon approach [43]. However, because sequencing libraries for this study required 1 μg of DNA, to generate sufficient starting material 1 μL of the mock community sample (5.5 ng of template, the amount recommended by the supplier for a typical reaction) was pre-amplified using Φ29 enzyme from the GenomiPhi V3 kit (25-6601-24, GE Healthcare Bio-Sciences, Pittsburgh, PA, USA) according to the manufacturer's recommendations. This version of Φ29 enzyme was chosen for isothermal pre-amplification due to the high-fidelity proof-reading aspects of its replication process [47].

The composition of each microbial mixture was calculated on the basis of the relative DNA mass contributed from each organism. Due to the random nature of shotgun sequencing, this library construction strategy is expected to result in a relative proportion of reads sequenced from each organism that corresponds to the relative input mass. In other words, the relative genome size of each organism should not have impacted the relative proportion of reads recovered from each organism.

Sequencing libraries were prepared for R7.3 flow cells run on an original MinION device using the Genomic DNA Sequencing Kit SQK–MAP005 (version 5 chemistry) according to the base protocol from Oxford Nanopore with slight modifications [48] and for flow cells run using the Nanopore Sequencing Kit SQK–MAP006 (version 6 chemistry) according to the manufacturer's recommendations. The steps for library SQK–MAP005 preparation included in this order: shearing 1 μg in a Covaris g-TUBE (Covaris, Inc., Woburn, MA, USA) at 2,000 × g for 2 min, treatment with PreCR (New England Biolabs, Beverly, MA, USA), cleanup with 1X AMPure beads (Agencourt, Beckman Coulter, Brea CA, USA), end-repair with NEBNext End Repair Module (New England Biolabs), cleanup with 0.5X AMPure beads, dA-tailing with NEBNext dA-Tailing Module (New

England Biolabs), ligation to a cocktail of both the leader and hairpin sequencing adapters (Oxford Nanopore Technologies) using Blunt TA Ligase (New England Biolabs), cleanup using his-tag Dynabeads (Life Technologies, City, State, USA), and recovery of the pre-sequencing mix in 25 μL of Elution Buffer (Oxford Nanopore Technologies). After priming the flow cell with EP solution according to the manufacturer's recommendations, an initial 6 μL aliquot of the pre-sequencing mix (at 10-20 ng/μL) was combined with 141 μL EP Solution and 3 μL Fuel Mix and applied to the flow cell. Thereafter, at 6-8 hr intervals, additional pre-sequencing mix aliquots (held on ice) combined with EP Solution and Fuel Mix were added to the flow cell at times roughly coinciding with re-programmed pore "remux," which is a process that adjusts the bias voltage and mux channels to maximize yield performance. Modified scripts (J. Tyson, pers. comm.) caused the MinION device to perform four remux steps at 8 h intervals to maintain regular increases in data (Figure 4).

Steps for library SQK–MAP006 preparation included in this order: shearing in a Covaris g-TUBE (Covaris, Inc., Woburn, MA, USA) at 2,000 × g for 2 min, treatment with PreCR (New England Biolabs, Beverly, MA, USA), cleanup with 1X AMPure beads (Agencourt, Beckman Coulter, Brea CA, USA), combined end-repair and dA-tailing with NEBNext UltraII End Repair/dA-Tailing Module (New England Biolabs), cleanup with 1X AMPure beads, ligation to a cocktail of both the leader and hairpin sequencing adapters (Oxford Nanopore Technologies) using Blunt TA Ligase (New England Biolabs), addition of a tether to the hairpin segment, cleanup using MyOne Streptavidin C1 Beads (Life Technologies, Carlsbad, CA, USA), and recovery of the pre-sequencing mix in 25 μL of Elution Buffer (Oxford Nanopore Technologies). After priming the flow cell with running buffer and fuel according to the manufacturer's recommendations, an initial 6 μL aliquot of the pre-sequencing mix (at 10-20 ng/μL) was combined with 75 μL Running Buffer, 65 μL water, and 4 μL Fuel Mix and applied to the flow cell. Thereafter, at 8 hr intervals, additional pre-sequencing mix aliquots (held on ice) were combined with Running Buffer and Fuel Mix and added to the flow cell at times roughly coinciding with re-programmed pore remux (modified scripts from J. Tyson, pers. comm.) Modified remux scripts were not used for the final MinION run (Staggered community analysis) because that run was controlled by a new version of MinKNOW.

Whole genome sequence data (2D FASTQ) from the MinION R7.3 flow cells were accessed on the MG-RAST server [23] and annotated based on their predicted proteins and rRNA genes using the BLAT annotation algorithm [49] against the M5NR protein Db, screened to remove any sequences matching *H. sapiens* (none found), and without dereplication or dynamic trimming. Although optimized for short read data, the MG-RAST tools were implemented because they allow query of a suite of comprehensive nonredundant genetic databases and because this server provides a means to share both raw data and computational results. Raw read counts were later accessed from MG-RAST using the API endpoint for organism summaries. The recommended parameters “hit_type=single”, “source=RefSeq”, and “evalue=15” were used to generate the appropriate read-level abundance information. The same read sets (2D FASTA) also were analyzed by Kraken [25] using the default k-mer size, minimizers, and other parameters, and accessing a local database created from archaea, bacteria, fungi, virus, protozoa, human, and invertebrate genomes. The Kraken tool was implemented because it is much faster than MG-RAST and allowed use of a smaller, more targeted reference database. The results were translated (kraken-translate) and summarized (kraken-report) to provide full taxonomic names for each classified sequence. Metagenomic analysis using One Codex was performed by uploading the 2D FASTQ data to the One Codex platform at https://app.onecodex.com. This cloud-based k-mer method was selected because it is reportedly more accurate than either the MG-RAST or the Kraken tools and because like MG-RAST, it provides for community access to the data and analytical results. Because of the high error rate of the R7.3 version MinION nucleotide data, the unfiltered One Codex results were used for this analysis, which do not include an automated error-filtering step. The One Codex read-level classification results were accessed by selecting the “unfiltered” option in the web-based results display and downloading a data table for each sample to generate appropriate read-level abundance information for tabulation.

Comparative data sets were generated for each of the four single species templates using full length ~1500 bp Sanger sequencing of a 16S amplicons [50]. Reads from the 16S analysis were subjected to BlastN for taxonomic assignment.

Figure 1 Result of "What's in my pot" analysis of a mixture with equal DNA mass from four bacterial strains.

Rendering of real-time analysis using WIMP [20] of whole genome sequences from a synthetic mixture prepared from equal DNA quantities of four cultured microbe species (experiment "Equal (6)" in Tables 1 and 2) and run on the MinION™ sequencing platform. Arc angle is proportional to the number of reads assigned to the indicated species. Colors (scale at bottom of diagram) refer to the classification score threshold (for this analysis the threshold for inclusion was 0.01).

Figure 2 Principal component analysis of normalized 5-mer frequency (i.e., percentage) within each MinION™ read for a mixture with equal DNA mass from four bacterial strains and a mixture with one rare component.

A: Sequencing run with equal DNA mass from four species. B: sequencing run with three equally represented (33% DNA mass each) and one rare (1% DNA mass) species included in the DNA pool. "none": read had no BlastN hits. "other": read had BlastN hits but not one of the four species included in the mix.

Figure 3 Log abundance of reads assigned from staggered mixture.

DNA of 20 species mixed in various proportions (BEI Resources, ATCC, HM-783D, operon counts μL^{-1} in original mixture indicated along bottom margin of bars) was pre-amplified with Φ29 polymerase prior to library preparation and sequenced with MinION™ R7.3 flow cells. The 2D reads that passed quality filtering were assigned to taxa using Kraken. Colored bars are species included in the mix whereas gray bars indicate species detected but not included in the original DNA mixture.

Figure 4 Read production using a MinION™ device and an R7.3 flow cell.

Illustration of reads collected from a synthetic metagenome made with equal DNA mass from four microbias species and a library prepared using SQK–MAP006 kit. Arrows indicate approximate times when additional aliquots of library and fuel were added.

Availability and requirements

- Project name: Experimental Metagenome on MinION
- Project home page: https://github.com/gigascience/paper link will be here.
- Operating system: Unix
- Programming language: Bash and R
- Other requirements: Unix
- License: N/A

Availability of supporting data

The datasets supporting the results of this article are available in the GigaDB repository [19], on the MG-RAST server 4629367.3, 4629445.3, 4629369.3, 4629381.3, 4614572.3, 4685746.3, 4685745.3, 4705090.3, and at the European Nucleotide Archive as primary accessions PRJEB8672 and PRJEB8716. One Codex results are available at https://app.onecodex.com/projects/bb_minion_env.

Competing interests

BLB, MW, MCR, and RBF are enrolled in the Oxford Nanopore MinION™ Access Programme (MAP) and received free materials for this research. SSM is an employee of One Codex.

Authors' contributions

BLB conceived of the study, performed the DNA extraction and sequencing, directed the data analysis, and drafted the manuscript. MW provided bioinformatic analyses and statistical analyses. MCR participated in study design, sequence alignment, and bioinformatic analysis. RBF participated in study design, sequencing, data analysis, and manuscript preparation. SSM performed some of the bioinformatic analyses and data interpretation. All authors read and approved the final manuscript.

Acknowledgements

This work was supported by the Virginia Commonwealth University Department of Biology (BLB, MCR, RBF), and by GenEco, LLC, Richmond, Virginia to BLB. Funding for MW was from the Biotechnology and Biological Sciences Research Council including Institute Strategic Programme and National Capability grants (BBSRC; BBS/E/D/20310000, BB/J004243/1, BB/M020037/1). The authors acknowledge M. Kensey Barker (VCU) for assistance with culturing bacteria. John Tyson (UBC) provided runtime plots and wrote the python scripts used to control the MinION device during the run. Hugh Eaves (VCU) provided programming assistance. Sarah Highlander (Venter Inst.) provided advice on determining DNA concentration. Michael Micorescu (ONT) provided assistance with Kraken. Arwyn Edwards and Kevin Keegan provided thorough review and suggestions for improvement of the manuscript. The following reagent was obtained through BEI Resources, NIAID, NIH as part of the Human Microbiome Project: Genomic DNA from Microbial Mock Community B (Staggered, Low Concentration), v5.2L, for 16S rRNA Gene Sequencing, HM-783D.

List of abbreviations

MAP: MinION™ Access Programme

2D: refers to sequences where both the template and the complement were completed (bidirectional) and passed the Metrichor quality threshold (Q9)

PCA: principal components analysis

gDNA: genomic DNA isolates from putatively pure cultures of bacterial strains

References

1. Mendoza MLZ, T Sicheritz-Ponten, MTP Gilbert. Environmental genes and genomes: understanding the differences and challenges in the approaches and software for their analyses. *Briefings in Bioinformatics*, 2015, 1–14. doi: 10.1093/bib/bbv001

2. Thomas T, Gilbert J, Meyer F. Metagenomics – a guide from sampling to data analysis. Microb Inform Experim. 2012;2:3.

3. Kasianowicz JJ, Brandin E, Branton D, Deamer DW. Characterization of individual polynucleotide molecules using a membrane channel. Proc Natl Acad Sci. 1996;93:13770–73.

4. Eisenstein M. Oxford Nanopore announcement sets sequencing sector abuzz. Nat Biotechnol. 2012;30:295–6.

5. Loman NJ, Quick J, Simpson JT. A complete bacterial genome assembled de novo using only nanopore sequencing data. Nat Methods. 2015;12:733–5.

6. Goodwin S, Gurtowski J, Ethe-Sayers S, Deshpande P, Schatz M, McCombie WR. Oxford Nanopore sequencing and de novo assembly of a eukaryotic genome. Genome Res. 2015a;25:1-7.

7. Risse J, Thomson M, Blakely G, Koutsovoulos G, Blaxter M, Watson M. A single chromosome assembly of Bacteroides fragilis strain BE1 from Illumina and MinION nanopore sequencing data. GigaScience 2015;4:60.

8. Quick J, Ashton P, Calus S, Chatt C, Gossain S, et al. Rapid draft sequencing and real-time nanopore sequencing in a hospital outbreak of *Salmonella*. Genome Biol. 2015;16:114.

9. Madoui M-A, Engelen S, Cruaud C, Belser C, Bertrand L, et al. Genome assembly using nanopore-guided long and error-free DNA reads. BMC Genomics. 2015;16:327.

10. Mongan AE, Yusuf I, Wahid I, Hatta M. The evaluation on molecular techniques of reverse transcription loop-mediated isothermal amplification (RT-LAMP), reverse transcription polymerase chain reaction (RT-PCR), and their diagnostic results on MinION™ nanopore sequencer for the detection of dengue virus serotypes. Am J Microbiol Res. 2015;3:118-24.

11. Hargreaves AD, Mulley JF. Snake venom gland cDNA sequencing using the Oxford nanopore MinION portable DNA sequencer. PeerJ. 2015 Nov 24;3:e1441. doi: 10.7717/peerj.1441. eCollection 2015.

12. Bolisetty MT, Rajadinakaran G, Graveley BR. Determining exon connectivity in complex mRNAs by nanopore sequencing. Genome Biol. 2015;16:204.

13. Cao MD, Ganesamoorthy D, Elliott A, Zhang H, Cooper M, Coin L. Streaming algorithms for identification of pathogens and antibiotic resistance potential from real-time MinION sequencing. bioRxiv 2015; doi: http://dx.doi.org/10.1101/019356.

14. Judge K, Harris SR, Reuter S, Parkhill J, Peacock SH. Early insights into the potential of the Oxford Nanopore MinION for the detection of antimicrobial resistance genes. J Antimicrob Chemother 2015; doi:10.1093/jac/dkv206.

15. Wang J, Moore NE, Deng Y-M, Eccles DA, Hall RJ. MinION nanopore sequencing of an influenza genome. Front Microbiol. 2015;6:766.

16. Kilianski A, Haas JL, Corriveau EJ, Liem AT, Willis KL, et al. Bacterial and viral identification and differentiation by amplicon sequencing on the MinION nanopore sequencer. GigaScience 2015;4:12.

17. Greninger AL, Naccache SN, Federman S, Yu G, Mbala P, et al. Rapid metagenomic identification of viral pathogens in clinical samples by real-time nanopore sequencing analysis. Genom Med. 2015;7:99.

18. Edwards A, Debbonaire AR, Sattler B, Mur LAJ, Hodson AJ. Extreme metagenomics using nanopore DNA sequencing: a field report from Svalbard, 78 °N. 2016. bioRxiv. doi: http://dx.doi.org/10.1101/073965

19. Brown, B, L; Watson, M; Minot, S, S; Rivera, M, C; Franklin, R, B (2017): Supporting data for "MinION nanopore sequencing of environmental metagenomes: a synthetic approach" GigaScience Database. http://dx.doi.org/10.5524/100278

20. Watson M, Thomson M, Risse J, Talbot R, Santoyo-Lopez J, et al. poRe: an R package for the visualization and analysis of nanopore sequencing data. Bioinformatics. 2015;31:114-5.

21. Loman NJ, Quinlan AR. Poretools: a toolkit for analyzing nanopore sequence data. Bioinformatics. 2014; 30:3399–3401.

22. Leggett RM, Heavens D, Caccamo M, et al.: NanoOK: Multi-reference alignment analysis of nanopore sequencing data, quality, and error profiles. Bioinformatics. 2015;32:142-4.

23. Meyer F, Paarmann D, D'Souza M, Olson R, Glass EM, et al. The metagenomics RAST server – a public resource for the automatic phylogenetic and functional analysis of metagenomes. BMC Bioinfo. 2008;9:386.

24. Juul S, Izquierdo F, Hurst A, Dai X, Wright A, Kulesha E, Pettett R, Turner D. What's in my pot? Real-time species identification on the MinION™. bioRxiv. 2015; doi: http://dx.doi.org/10.1101/030742.

25. Wood DE, Salzberg SL. Kraken: ultrafast metagenomic sequence classification using exact alignments. Genome Biol. 2014;15:R46.

26. Minot SS, Krumm N, Greenfield NB. One Codex: a sensitive and accurate data platform for genomic microbial identification. bioRxiv. 2015. doi: http://dx.doi.org/10.1101/027607

27. Altschul SF, Madden TL, Schaffer AA, Zhang J, Zhang Z, Miller W, Lipman DJ. Gapped BLAST and PSI-BLAST: a new generation of protein database search programs. Nucl Acids Res. 1997;25:3389.

28. R Core Team. R: A language and environment for statistical computing. R Foundation for Statistical Computing, Vienna, Austria. 2015;https://www.R-project.org/.

29. Park H-D, Sasaki Y, Maruyama T, Yanagisawa E, Hiraishi A, Kato K. Degradation of the cyanobacterial hepatotoxin microcystin by a new bacterium isolated from a hypertrophic lake. Environ Toxicol 2001;16:337-43.

30. Jones MB, Highlander SK, Anderson EL, Li W, Dayrit M, et al. Library preparation methodology can influence genomic and functional predictions in human microbiome research. Proc Nat Acad Sci. 2015;45:14024-9.

31. Wommack KE, Bhavsar J, Ravel J. Metagenomics: Read Length Matters. Appl Environ Microbiol. 2008;74:1453.

32. Brown BL, LePrell RV, Franklin RB, Rivera MC, Cabral FC, et al. Metagenomic analysis of planktonic microbial consortia from a non-tidal urban-impacted segment of James River. Stand Genomic Sci. 2015;10:65.

33. Magasin JD, Gerloff DL. Pooled assembly of marine metagenomic datasets: enriching annotation through chimerism. Bioinformatics. 2015;31:311-317.

34. Freitas TAK, Li P-E, Scholz MB, Chain PSG. Accurate read-based metagenome characterization using a hierarchical suite of unique signatures. Nucl Acids Res. 2015; doi: 10.1093/nar/gkv180.

35. Zhang Q, Ye Y, Doak TG. Artificial functional difference between microbial communities caused by length difference of sequencing reads. Biocomputing 2012;259-270. DOI: http://dx.doi.org/10.1142/9789814366496_0025.

36. Frank JA, Pan Y, Tooming-Klunderud A, Eijsink VGH, McHardy AC, Nederbragt AJ, Pope PB. Improved metagenome assemblies and taxonomic binning using long-read circular consensus sequence data. Sci Rep. 2016:6:25373 doi:10.1038/srep25373.

37. Sović I, Šikić M, Wilm A, Fenlon SN, Chen S, Nagarajan N. Fast and sensitive mapping of nanopore sequencing reads with GraphMap. Nat Comm. 2016;11307. doi:10.1038/ncomms11307 doi:10.1038/ncomms11307.

38. Chaisson MJ, Tesler G. Mapping single molecule sequencing reads using basic local alignment with successive refinement (BLASR): application and theory. BMC Bioinfo. 2012;13:238 doi: 10.1186/1471-2105-13-238.

39. Hugh R. Note: *Pseudomonas maltophilia* sp. nov., nom. rev. Int J System Evol Microbiol. 1981;31:195. doi: 10.1099/00207713-31-2-195

40. Binga EK, Lasken RS, Neufeld JD. Something from (almost) nothing: the impact of multiple displacement amplification on microbial ecology. The ISME Journal. 2008;2:233-241.

41. Lasken RS, Stockwell TB. (2007). Mechanism of chimera formation during the Multiple Displacement Amplifi- cation reaction. BMC Biotechnol 7: 19. doi:10.1186/1472-6750-7-19

42. Li C, Chng KR, Boey EJH, Ng AHQ, Wilm A, Nagarajan N. INC-Seq: accurate single molecule reads using nanopore sequencing. GigaScience. 2016;5:34.

43. Benítez-Páez A, Portune KJ, Sanz Y. Species-level resolution of 16S rRNA gene amplicons sequenced through the MinION™ portable nanopore sequencer. GigaScience. 2016;5:4.

44. Goodwin S, Gurtowski J, Ethe-Sayers S, Deshpande P, Schatz M, McCombie WR. Oxford Nanopore sequencing, hybrid error correction, and *de novo* assembly of a eukaryotic genome. Genome Res. 2015b;25:1750-6.

45. Karlsson E, Lärkeryd A, Sjödin A, Forsman M, Stenberg P. Scaffolding of a bacterial genome using MinION nanopore sequencing. Scientif Rep 2015;5:11996.

46. Stephens ZD, Lee SY, Faghri F, Campbell RH, Zhai C, et al. Big data: astronomical or genomical? PLoS Biol. 2015;13:e1002195. doi:10.1371/journal.pbio.1002195.

47. Garmendia C, Bernad A, Esteban JA, Blanco L, Salas M. The bacteriophage phi 29 DNA polymerase, a proofreading enzyme. J Biol Chem. 1992;267: 2594-2599.

48. Ip CLC, Loose M, Tyson JR, Cesare M, Brown BL, et al. MinION Analysis and Reference Consortium: Phase 1 data release and analysis. F1000Research 2015;4:1075.

49. Kent WJ. BLAT—the BLAST-like alignment tool. Genome Res. 2002;12:656–64.

50. Bartram AK, Lynch MD, Stearns JC, Moreno-Hagelsieb G, Neufeld JD. Generation of multimillion-sequence 16S rRNA gene libraries from complex microbial communities by assembling paired-end Illumina reads. Appl Environ Micro. 2011;77:3846–52.

Figure1

Click here to download Figure Figure1BrownetalMinIONMetagenome.docx

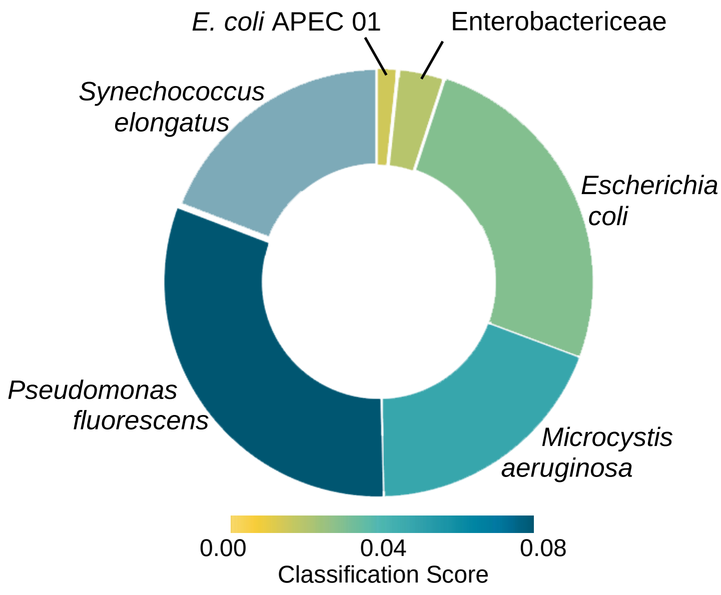

Figure2

Click here to download Figure Figure2BrownetalMinIONMetagenome.docx

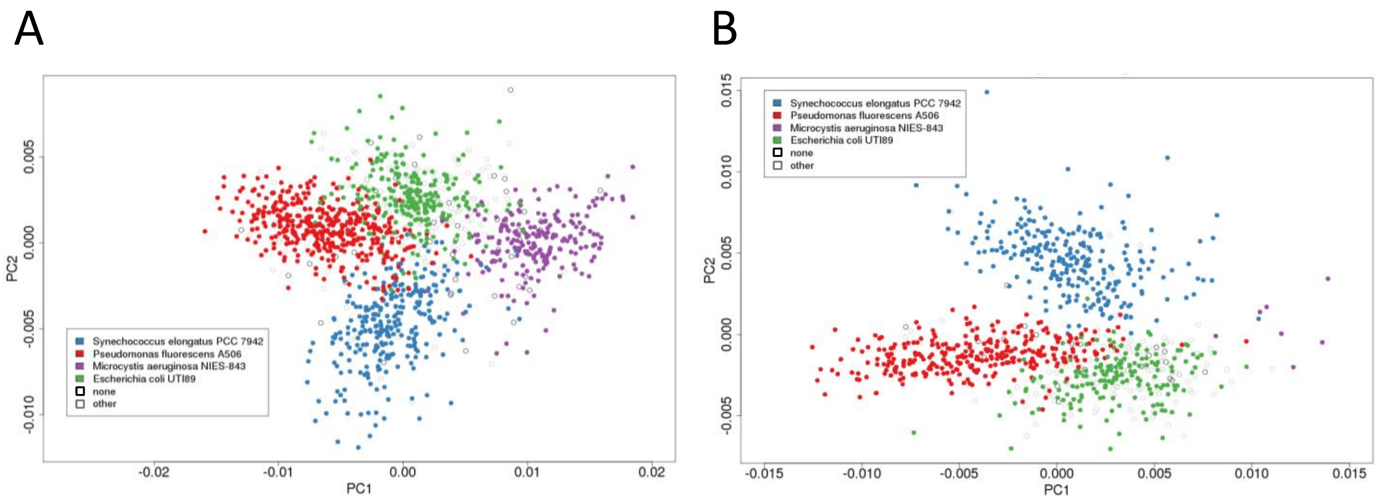

Figure3

Click here to download Figure Figure3BrownetalMinIONMetagenome.docx

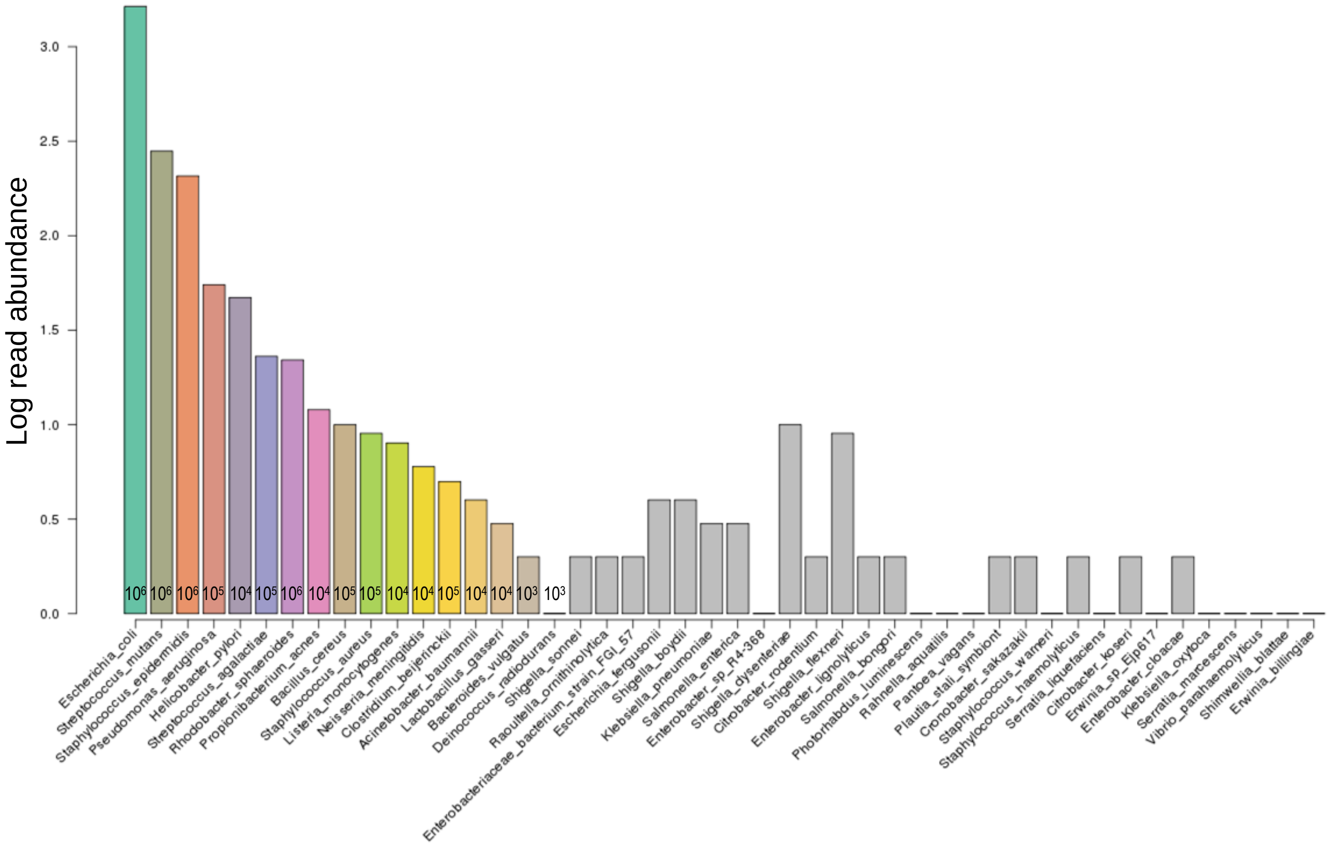

Figure4

Click here to download Figure Figure4BrownetalMinIONMetagenome.docx

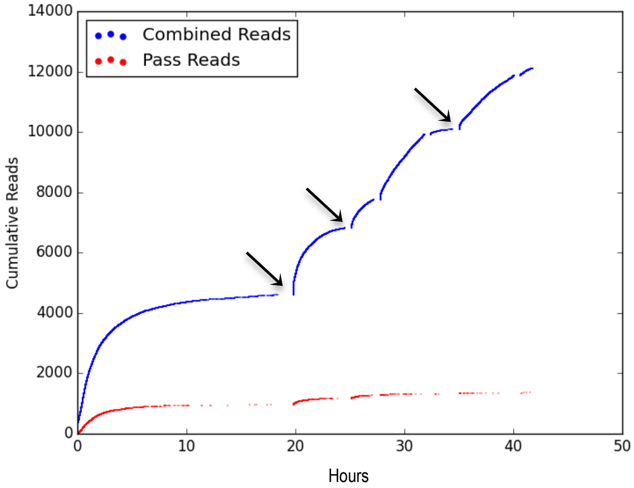

Supplement: GIGA-D-16-00081_Revision_2.pdf [file gix007_GIGA-D-16-00081_Revision_2.pdf]
