# Supplementary material for: MinION™ nanopore sequencing of environmental metagenomes: a synthetic approach
Source: Gigascience. 2017 Feb 24;6(3):1–10. doi: 10.1093/gigascience/gix007 (PMC5467020; doi:10.1093/gigascience/gix007)
Supplement: Response_to_Reviewer_Comments_Original_Submission.pdf [file gix007_Response_to_Reviewer_Comments_Original_Submission.pdf]

Reviewer #1:

Summary

The authors present their results from the application of a nanopore-based sequencing device, the MinION from Oxford Nanopore Technologies, to samples containing DNA from known bacterial organisms. Organisms from four distinct genera are included as well as a mixture consisting of 20 distinct organisms at staggered concentrations. As such, the authors demonstrate the basic applicability of MinION-based sequencing for the study of mixed microbial communities, which I consider very relevant to the field of metagenomics. While I would have liked to see additional computational approaches, especially those made for high-error sequences, used in this study, the presented results suggest that species-level identification and quantification are possible, albeit with some (expected) variation.

General comments

- The authors seem to have used two different models of the MinION sequencing device, a "pre-full production" model and the Mk1, i.e., a full production model. This could be made more explicit in general and further expanded on in the respective specific cases throughout the manuscript. Readers which are less experienced with the "deployment strategies" of Oxford Nanopore Technologies might be more easily confused otherwise.

Revised throughout to clarify that all flow cells were R7.3 and to better indicate the chemistry.

- It is nice to see that the authors used a set of different (taxonomic) analysis tools, in particular tools which are commonly used for the purpose of analysising metagenomic data. However, I was missing tools which were especially designed for long read data which is currently still characterized by high sequencing error rates. Examples of such tools are BLASR or DALIGNER. These tools might lead to improved taxonomic assignments. Moreover, for Kraken, varying the size of the k-mers which are used to construct the reference database could have an important effect on the assignments as one sequencing error will affect k consecutive k-mers. Furthermore, Kraken offers various ways of assigning taxonomy to a read. Using the quick mode, a single matching k-mer is sufficient to assign taxonomy to a read. This is expected to be the least robust way of taxonomic assignment. In the default mode, a Lowest Common Ancestor-based approach is used. However, should the number of k-mer hits be low, this should also be treated with caution. Finally, Kraken offers a "filter" mode based on "confidence scoring" which will give more precise results at the price of reduced sensitivity. While it may be difficult to perform (all of) these or similar experiments, I think they should at least be discussed.

We have added a results of analysis using a new tool One Codex that we hope satisfies this concern.

- More information should be provided in the Methods section about the way the individual tools were used, e.g., whether the used Kraken-database was built using the default parameters or if potentially a smaller value was used for k. While I am not sure whether this is true, it seems that WIMP used a k-mer size of 24 ("ver WIMP Bacteria k24 for SQK-MAP006" in L121-122).

We added the conditions for each analysis type in the Methods section.

Specific comments

- L30: I found the formulation of this very first sentence somewhat confusing. Specifically, it read like taxonomy is typically achieved based on amplicon sequencing but not by WGS. Thus, I would recommend rephrasing this sentence.

Sentence changed

- L42: Throughout this study, only R7.3 flow cells were used. Hence, I would suggest to highlight this once in the abstract but not to repeat it in the abstract as it represents redundant information.

All instances of the flow cell version have been either eliminated or clarified

- L56: I am not sure whether the mock metagenome that was sequenced in this study qualifies as an "environmental metagenome" in the "traditional" sense, i.e., derived from an actual environmental sample. Accordingly, I would suggest removing the "environmental" from this sentence and replace it, e.g., "environmental consortium" "mixed microbial community" or something along those lines.

Phrasing changed to “synthetic metagenome” and “where the consortium is comprised”

- Coinciding with the present work, a preprint by Edwards et al. (http://biorxiv.org/content/early/2016/09/07/073965) was published in which the authors sequenced actual

environmental samples to characterize the microbiota of a High Arctic glacier. Since both of the studies, the current work and the work of Edwards et al. are among the first to apply the MinION sequencer to metagenomic samples, it might be good to include a reference to the preprint in the current work.
Mention has been made in several places of this important work.
Moreover, while Kraken is expected to be much faster than BLASTN, the percent-values in Table 2 did not show that either of these two approaches performed substantially better throughout but rather that BLASTN was "better" than Kraken in some cases and vice versa. Clearly, both approaches outperformed MG-RAST-based analyses, which is something that could be highlighted and discussed (more).
BLASTN is no longer included in the comparison (although results will be available on the site and are used to annotate the PCA) and the relative performance of MG, Kraken, and One Codex are now compared in the Results and Discussion.
- L62: While I agree with the authors that for some of the studied samples, high percentages of correct taxonomic assignments are reached, the current formulation of the sentence might be misleading. There is, expectedly, quite some variation in the assignment percentages reported, e.g., 88.2% for the equal mixture (5) and BLASTN. Moreover, the largest percent-value in Table 2 is 96.6% which is < 97%. Thus, this should be rephrased. One way would be to report the mean +- sigma of the best performances across all tools or for Kraken or BLASTN.
Sentence rewritten to reflect this observation.
- L86 - 95: I find that this paragraph could benefit from supporting references, e.g., for the metagenome-based study of the functional potential of mixed microbial communities.
Thanks for catching this oversight, we added references.
- L96: There might also be "short" contigs, hence I would suggest omitting the "large" or put it in parentheses.
Deleted “large”
- L99: Maybe provide a reference for the "high likelihood of generating chimeric contigs".
Reference added
- L99 - 100: I am not sure about the "there is no chance of chimeras" if there is no assembly involved. The likelihood is surely much lower but couldn't it happen during some amplification steps potentially? While I would rephrase this first part of the sentence, I agree with the rest of it.
Added “Absent a preamplification step, if WGS reads are not assembled,…”
- L124 - 125: It reads as if something is missing before the "archive". Maybe "and only the 2D reads were archived/stored/extracted into FASTQ and FASTA files ..."?
Sentence corrected
- L126: While I agree that BLASTN, Kraken, and MG-RAST are commonly used in metagenomic data analysis, they are much less "common" for the analysis of long read sequencing data. After all, long reads currently still contain a rather high proportion of sequencing errors. Computational tools which account for this higher error rate might lead to improved results, see also my general comments on this point.
Sentence changed to reflect this distinction
- L129: An analysis approach that relies on in-silico translated protein sequences might be particularly challenged by high-error data due to the incurred challenges of detecting start and stop-codons and/or to identify the "correct" amino acid. I feel that this could be made more explicit in the Discussion. Moreover, while I am hesitant to suggest to perform more experiments, using a different protein sequence-based tool, e.g., DIAMOND, could be very interesting to be included as that would then total to two DNA sequence-based approaches and two protein sequence-based approaches.
The challenges inherent in using MG-RAST are better outlined now in the Discussion
- L142: Table 1 gives a summary of the results. As such, it can not be used in its current form to read out information such as "5 bp to as long as 267x10^3 bp".
Sentence changed
- L143: P. fluorescens hast only 79.3% assignment in Table 2. Is this correct or is this an artefact in Table 2? In either case, this should be verified and adjusted accordingly, either in the text or in the table.
Table and relevant text corrected
- L143 - 144: The authors refer to "species", yet Table 2 refers to genus-level assignments. This should be clarified.
Table is species level assignment so the table title has been corrected.

- L152: Please see my comment on L142 above.
Sentence changed
- L154: It was not readily clear to me what the authors meant by "early" here. It seems as if this refers to the use of the original MinION device, as opposed to the Mk1. Thus, I suggest to clarify this point.
Correct, the paragraph has been edited to clarify.
- L161: "Read annotation of 5-mers" reads strangely to me. Based on my understanding, the reads were assigned to a specific taxon and for each read using BLASTN, the 5-mer frequency profiles were computed, and then visualized. I would thus suggest rephrasing this sentence.
Sentence revised
- L164: It is nice to see that an unsupervised approach was used to inspect whether meaningful cluster structures would be apparent despite the high sequence error rates. However, PCA is probably not the most sensitive approach to this, albeit it is a commonly used approach for the purpose of dimension reduction. I would thus suggest to use approaches which were developed more recently, e.g., based on Emergent Self-Organizing Maps (ESOMs) or based on Barnes-Hut Stochastic Neighbor Embedding (BH-SNE). The latter is integrated into VizBin, which (full disclosure) I am the first author of. While I did not have the per-read taxonomic assignments readily available (only the BLASTN output), I performed my own experiment and used VizBin with the FASTA files of the "equal" and the "rare" datasets. In both cases, distinct clusters with less overlap than in the PCA plot were revealed. While this could be quantitatively evaluted using a 2D clustering approach, this is likely beyond the scope of this work. Moreover, from my personal experience, PCA plots for k-mer frequency profiles are likely to be much less informative if the number of clusters increases, while ESOM- or BH-SNE-based approaches have been shown to readily resolve cluster structures even for higher numbers of clusters. I would thus suggest to elaborate on this point and would be happy to provide further information to the authors if required.
As this data set and the R7.3 technology already are obsolete, we chose not to address this issue at the present time. We do however gratefully acknowledge this approach and are implementing it for a “real” data set generated using R9.x MinION technology. If the current graph is so impeachable as to render it useless, which we don’t this is true, we will remove it.
- L170: It seems that a verb is missing here, maybe "mapped" or "assigned"?
Sentence corrected
- L255: The use of "extra-species sequences" sounded a bit confusing to me in this context. I would consider linkers/primers/adapters as "extra-species", but not symbionts, parasites, or pathogens. Thus this should be clarified.
All of the types of sequences in our list are contaminants that have found their way into the public data base. We changed the sentence to read “…sequences that are not innate to the species that was analyzed; these include symbionts,…”
- L258-260: I found this sentence difficult to read. Please consider rephrasing it.
Sentence shortened and clarified
- L290-291: While I would also intuitively suspect more reference sequences for E. coli than for R. sphaeroides, I would suggest to provide concrete numbers here, e.g., the number of genomes for the respective species at the NCBI Taxonomy database.
Excellent point, sentence clarified
- L302-303: While I am also optimistic about further technological improvements in the field of long read sequencing, I found the last part of the sentence a bit to strong and would suggest toning the last part down or removing it as it is rather speculative.
Sentence changed to read “we speculate”
- L340: In my copy of the manuscript, there was apparently an encoding error with the "25 ?L of Elution Buffer". This is however hopefully fixed in a later version.
Now says 25 µL
- L357: Same problem as with L340.
Now says 25 µL
- L368: A stated above, the difference in the results between MG-RAST-based analyses and BLASTN/Kraken-based analyses could be discussed more. Here, i.e., in the Methods section, more details about the parameters used to run the various tools should be provided. Maybe, if not already planned, putting the analysis code (scripts, parameters, etc.) online would be good too.

More details of the parameters for running the various tools are now in the Methods section
- L380: Please include references to the panels (A and B) into the legend.
Added reference to the two panels.
- L395: Please clarify what "combined reads" and "pass reads" refer to.
Revised the legend for this figure
- Table 1: Please include some description as to why the runtime varies. Was it because the run simply ended, because the input material was completely consumed, or was the run simply terminated because of sufficient sequence data generated? Was any of the runs followed after a previous run, or were fresh flow cells used for each experiment? Please check the consistency of the capitalization in the table's headings.
Clarified in Methods that fresh flow cells were used for each run. Added a footnote below the table to clarify why run times varied (always fresh flow cells and run continued until sufficient sequence data were collected – or the 2D pass rate was greatly reduced). Now Table 2.
- Table 2: The legend refers to genus-level assignments, yet the main text refers to the species level. Please clarify this point.
Clarified in both the text and the table. Now Table 3.
- Table 3: The font was different from all the other tables. This is likely to be fixed in a later version.
Now Arial narrow
- Table 4: Please check the consistency of the capitalization in the table's headings, i.e., "species" vs. "Genus"
They both are now capitalized.

Reviewer #2:

The manuscript presents a benchmarking exercise to evaluate the use of the MinION based nanopore DNA sequencing platform to conduct shotgun metagenomics. The authors test MinION's ability to correctly assign reads from single and mixed species sequencing runs as well as a contrived/mock community. A particular strength of the manuscript is the comparison of different analysis approaches (MGRAST, Kraken, BLAST) which might be used to analyse nanopore metagenomes, rather than just one approach. I find their overall conclusions to be reasonable and well supported by their data. The manuscript is well written, and the experimental approaches suitable for the most part.

Within the manuscript there are some issues which need to be addressed. Principally these pertain to technical issues pertaining to the 20 species mock community sequenced.

1. The authors detail (L329) that the limited quantity of mock community DNA necessitated Phi29 preamplification to obtain 1 ug DNA for library preparation. Multiple displacement amplification presents a well-known bias in the analysis of low biomass samples by shotgun metagenomics, and in some cases Phi29 amplification can itself produce chimeric products (reviewed: Binga et al. 2008 ISMEJ doi:10.1038/ismej.2008.10). Considering the authors make a considerable virtue of the reduced potential for biases introduced via chimeric assemblies (L99) and amplification-based metagenomics (L59) presented by nanopore shotgun metagenomics this presents two sources of bias which are not well detailed within the manuscript. In a revision I believe the authors could justify their use of this particular mock community better, and discuss the potential errors introduced by the Phi29 amplification in the context of their evaluation of the mock community. While I am of the opinion that the generation of an ad hoc community from the admixture of higher concentration genomic DNAs would be a fairer test of the platform than this approach, considering a potential application in low-biomass / low complexity communities identified by the authors (L300) perhaps this approach is justified. Regardless, this segment of the manuscript would benefit from clearer justification and caveating of the experimental approach.
Justification for the choice of the BEI has been added to the methods. The chimeras resulting from Phi29 would not result in taxonomic ID failure, but they could possibly result in taxonomic over-representation. We described this in the Discussion. Also, thanks for sharing, we have added the reference.
2. Table 4 specifies both rRNA operon copy number and pg/uL DNA content. I would hope the latter reflects the actual genomic content of individual species within the mock community, but it is unclear. As the

authors are undoubtedly aware, rRNA operon copy number varies between taxa. The manuscript does not specify if/how this variation has been controlled for, given that the mock community has been staggered by rRNA operon copy number, rather than total genome content. This should be clarified, and if unaddressed, corrected for in the calculations of actual species proportions within the mock community.
Your interpretation is correct. We added superscripts in the header to clarify.
3. Handling obsolecence gracefully. By the time of submission R7 flow cells, sequencing chemistry and scripts outlined in this manuscript are no longer available, and as the authors highlight, the prospect of improved performance enhancing the potential for nanopore metagenomics is approaching. Nevertheless I believe the manuscript retains considerable value in setting out a baseline in nanopore metagenomics. In a revision, I believe the manuscript would benefit from clearly acknowledging the rapid advancement within the field in the interim, and presenting the caveat that the limitations described are likely to be surpassed in the near future, if not already. This might be achieved by expanding and updating the discussion at L308-312.
The Discussion has been expanded per this suggestion.
4. L145: "to closely related cyanobacteria genera, Sphingobacteriaceae" In what way is the family Sphingobacteriaceae within the Bacteroidetes phylum a closely related genus to the genus Microcystis within the phylum Cyanobacteria? This is a significant taxonomic error which renders the justification presented for mis-assignment of reads open to considerable question. In a revision this statement should be corrected and the underlying cause clearly requires further thought.
The statement had a terribly embarrassing error. The erroneous taxon has been deleted and replaced with the correct taxon.

Reviewer #3:

The authors conducted a study of using MinION nanopore sequencing to characterize environment metagenomes. The 2D long reads were used in attempt to assign taxa of different synthetic mixtures up to 20 species. Several analytical tools was applied for this purpose.

The manuscript is scientifically sound and basically well-written. The results show potential application of nanopore sequencing in the field of environment metagenomics, although the performances are still limited for the time beings.

There are some opinions from my point of view:

1. In Table 1, the unit for the fourth column (Total bp) must be Mbp instead of Gbp when it comes to the yield of a typical MinION run.
This error has been corrected in the header. Now Table 2.

2. Table 2 is quite difficult to follow and catch the point. I expect a graph for better visualization instead of numeric table in this circumstance. In addition, there is a significant deviation between results of using MG-RAST with the other two but I hardly found any discussion about this. It is also better if the authors could briefly review the 3 methods used in their study for community analysis and the reasons they were chosen in a bit more details.
We fixed the organization of the table (instead of making a graph) to make it easier to interpret. Now Table 3.

3. The authors claimed that using long reads has advantages over short read data for metagenomics studying. If applicable, a real comparison of such that case, e.g. using MinION vs Illumina data for community analysis, would make the point more convincing.
The justification for our paper is that there aren’t any data sets for MinION. We have included a recent citation for PacBio and data to illustrate this point.

Reviewer #4:

The authors report the study of three synthetic microbial communities using WGS in the portable DNA sequencer, MinION, as well as genome analysis of single species merged in such artificial communities. Although genome sequencing analysis can be found regularly in the scientific literature, the authors put special attention to the usage of MinION reads to perform environmental metagenomics, an approach of limited study by using third generation sequencing technologies, therefore this study compiles data and analyses of interest for future approaches regarding the better description of microbial communities in terms of species inventory and function as well. Common microbial community taxonomy and diversity analysis were used to evaluate the data produced by MinION device concluding a modest performance of MinION device.

In my opinion, this work represent an interesting workflow to help in the achievement of valuable information regarding the complete assessment of environmental microbial communities, where the limitations in terms of read length of the second generation of sequencing technologies constitute major issues concerning the species identification. As a consequence, I recommend this manuscript for publication in GigaScience journal once the following changes have been incorporated to the main text and supporting material:

Major concerns:

1. The authors claim in the introduction that "very short reads can lack information needed to properly identify the source of the read due to conservation of gene sequences across related organisms"; and given that in line 142 they describe that DNA reads obtained from single-species runs range from 5bp to hundreds of kbp, I miss a filtering step on MinION data to retain larger fragments (e.g. > 1 or >2 kb) previous to the taxonomy assessment. This could improve the results exhibited in the Table 2.
The results quoted were intended to illustrate the characteristics of all reads that were output from the device, both pass and fail. This has been clarified in the text and an additional statement has been added to clarify what reads were subjected to downstream analysis. For this reviewer's information, the smallest 2D pass read analyzed was 118bp, but the vast majority were >2000 bp.
2. Why the authors did not consider to use the "low-input" kit from ONT to perform the sequencing analysis of the mock staggered community?
These data were collected prior to the availability of the "low input" kit. In coordination with ONT, we did try LPCR but the results were terribly skewed and we decided not to include those in this paper as ONT no longer promotes that technique.
3. In line 154 the authors describe use of MinION device but two lines below describe to using the MkI device. Have the authors observed any difference in the performance of these both devices? Could this change of device affect in somehow the analysis?
The old and new devices optimally control the flow cells so we have modified the ms by removing reference to the Mk1 and have instead included better reference to the flow cells, software version, and the sequencing chemistry throughout.
4. In my opinion, the very low amount of 2D reads retrieved from the sequencing process is the major handicap of this technology to assess metagenomics from environmental- or human-derived samples (median of ~5% of all runs). The authors have to be more conclusive in this way and clearly state that unless performance in terms of number of reads be extremely increased, the MinION platform would not be appropriate to perform metagenomics of complex samples.
We agree and have included a statement to this effect in the Discussion.
5. The author must discuss in a deeply manner the findings regarding the annotation of metagenomic sequences derived from MinION. It is quite evidently that MG-RAST did not process the MinION data in a proper way and the algorithms behind every assignment tool should be compared to get consensus procedure that better fit to MinION data.
The choice of bioinformatic tool and the limitations of MG-RAST in particular have been better defined in the Methods and included in the Discussion.
6. Data coming from the analysis of the 20-species mock staggered community are disappointing and interesting at the same time. In one hand, WIMP seems to work very well in terms of the performance

needed for metagenomics analyses. Given that WIMP directly connect to NCBI database, it is able to compare MinION data with one of the largest DNA repositories offering reliable results at real-time regarding the composition of the microbial community under study. On the other hand, the strong bias at microbial composition level observed when the mock staggered community was analyzed could be also explained by the genome structure itself. The author should correlate the bias among the observed and expected composition of the microbial community for every single species with the GC content or other parameter of genome complexity. Additionally, the authors should measure the absolute abundance of certain species at the original mock staggered community and after the linear amplification with Phi29 polymerase by qPCR methods. This will be very helpful to shed light on the origin of such abundance bias.
All DNA concentrations were determined after Phi29 treatment. We apologize for confusion and have corrected the ms. We did not perform individual qPCR tests because both this data set and the R7.3 technology already are obsolete and because there was good evidence that the Phi29 treatment itself was the culprit. However, we do gratefully acknowledge this suggested approach and will implement it for future trials using R9.x MinION technology.
