# Supplementary material for: MinION™ nanopore sequencing of environmental metagenomes: a synthetic approach
Source: Gigascience. 2017 Feb 24;6(3):1–10. doi: 10.1093/gigascience/gix007 (PMC5467020; doi:10.1093/gigascience/gix007)
Supplement: Response_to_reviewer_comments_Revision_1.pdf [file gix007_Response_to_reviewer_comments_Revision_1.pdf]

All changes suggested by Reviewer #1 have been made.
