# Supplementary material for: MinION™ nanopore sequencing of environmental metagenomes: a synthetic approach
Source: Gigascience. 2017 Feb 24;6(3):1–10. doi: 10.1093/gigascience/gix007 (PMC5467020; doi:10.1093/gigascience/gix007)
Supplement: Reviewer_1_Report_(Original_Submission).pdf [file gix007_Reviewer_1_Report_(Original_Submission).pdf]

Reviewer Report

Title: "MinION™ nanopore sequencing of environmental metagenomes: a synthetic approach"

Version: Original Submission **Date:** 9/13/2016

Reviewer name: Cedric Laczny, Ph.D.

Reviewer Comments to Author:

Summary

The authors present their results from the application of a nanopore-based sequencing device, the MinION from Oxford Nanopore Technologies, to samples containing DNA from known bacterial organisms. Organisms from four distinct genera are included as well as a mixture consisting of 20 distinct organisms at staggered concentrations. As such, the authors demonstrate the basic applicability of MinION-based sequencing for the study of mixed microbial communities, which I consider very relevant to the field of metagenomics. While I would have liked to see additional computational approaches, especially those made for high-error sequences, used in this study, the presented results suggest that species-level identification and quantification are possible, albeit with some (expected) variation.

General comments

- The authors seem to have used two different models of the MinION sequencing device, a "pre-full production" model and the Mk1, i.e., a full production model. This could be made more explicit in general and further expanded on in the respective specific cases throughout the manuscript. Readers which are less experienced with the "deployment strategies" of Oxford Nanopore Technologies might be more easily confused otherwise.

- It is nice to see that the authors used a set of different (taxonomic) analysis tools, in particular tools which are commonly used for the purpose of analysising metagenomic data. However, I was missing tools which were especially designed for long read data which is currently still characterized by high sequencing error rates. Examples of such tools are BLASR or DALIGNER. These tools might lead to improved taxonomic assignments. Moreover, for Kraken, varying the size of the k-mers which are used to construct the reference database could have an important effect on the assignments as one sequencing error will affect k consecutive k-mers. Furthermore, Kraken offers various ways of assigning taxonomy to a read. Using the quick mode, a single matching k-mer is sufficient to assign taxonomy to a read. This is expected to be the least robust way of taxonomic assignment. In the default mode, a Lowest Common Ancestor-based approach is used. However, should the number of k-mer hits be low, this should also be treated with caution. Finally, Kraken offers a "filter" mode based on "confidence

scoring" which will give more precise results at the price of reduced sensitivity. While it may be difficult to perform (all of) these or similar experiments, I think they should at least be discussed.

- More information should be provided in the Methods section about the way the individual tools were used, e.g., whether the used Kraken-database was built using the default parameters or if potentially a smaller value was used for k. While I am not sure whether this is true, it seems that WIMP used a k-mer size of 24 ("ver WIMP Bacteria k24 for SQK-MAP006" in L121-122).

Specific comments

- L30: I found the formulation of this very first sentence somewhat confusing. Specifically, it read like taxonomy is typically achieved based on amplicon sequencing but not by WGS. Thus, I would recommend rephrasing this sentence.

- L42: Throughout this study, only R7.3 flow cells were used. Hence, I would suggest to highlight this once in the abstract but not to repeat it in the abstract as it represents redundant information.

- L56: I am not sure whether the mock metagenome that was sequenced in this study qualifies as an "environmental metagenome" in the "traditional" sense, i.e., derived from an actual environmental sample. Accordingly, I would suggest removing the "environmental" from this sentence and replace it, e.g., "environmental consortium" -> "mixed microbial community" or something along those lines. Coninciding with the present work, a preprint by Edwards et al. (http://biorxiv.org/content/early/2016/09/07/073965) was published in which the authors sequenced actual environmental samples to characterize the microbiota of a High Arctic glacier. Since both of the studies, the current work and the work of Edwards et al. are among the first to apply the MinION sequencer to metagenomic samples, it might be good to include a reference to the preprint in the current work. Moreover, while Kraken is expected to be much faster than BLASTN, the percent-values in Table 2 did not show that either of these two approaches performed substantially better throughout but rather that BLASTN was "better" than Kraken in some cases and vice versa. Clearly, both approaches outperformed MG-RAST-based analyses, which is something that could be highlighted and discussed (more).

- L62: While I agree with the authors that for some of the studied samples, high percentages of correct taxonomic assignments are reached, the current formulation of the sentence might be misleading. There is, expectedly, quite some variation in the assignment percentages reported, e.g., 88.2% for the equal mixture (5) and BLASTN. Moreover, the largest percent-value in Table 2 is 96.6% which is < 97%. Thus, this should be rephrased. One way would be to report the mean +- sigma of the best performances across all tools or for Kraken or BLASTN.

- L86 - 95: I find that this paragraph could benefit from supporting references, e.g., for the metagenome-based study of the functional potential of mixed microbial communities.

- L96: There might also be "short" contigs, hence I would suggest omitting the "large" or put it in parentheses.

- L99: Maybe provide a reference for the "high likelihood of generating chimeric contigs".

- L99 - 100: I am not sure about the "there is no chance of chimeras" if there is no assembly involved. The likelihood is surely much lower but couldn't it happen during some amplification steps potentially? While I would rephrase this first part of the sentence, I agree with the rest of it.

- L124 - 125: It reads as if something is missing before the "archive". Maybe "and only the 2D reads were archived/stored/extracted into FASTQ and FASTA files ..."?

- L126: While I agree that BLASTN, Kraken, and MG-RAST are commonly used in metagenomic data analysis, they are much less "common" for the analysis of long read sequencing data. After all, long reads currently still contain a rather high proportion of sequencing errors. Computational tools which account for this higher error rate might lead to improved results, see also my general comments on this point.

- L129: An analysis approach that relies on in-silico translated protein sequences might be particularly challenged by high-error data due to the incurred challenges of detecting start and stop-codons and/or to identify the "correct" amino acid. I feel that this could be made more explicit in the Discussion. Moreover, while I am hesitant to suggest to perform more experiments, using a different protein sequence-based tool, e.g., DIAMOND, could be very interesting to be included as that would then total to two DNA sequence-based approaches and two protein sequence-based approaches.

- L142: Table 1 gives a summary of the results. As such, it can not be used in its current form to read out information such as "5 bp to as long as 267x10^3 bp".

- L143: P. fluorescens hast only 79.3% assignment in Table 2. Is this correct or is this an artefact in Table 2? In either case, this should be verified and adjusted accordingly, either in the text or in the table.

- L143 - 144: The authors refer to "species", yet Table 2 refers to genus-level assignments. This should be clarified.

- L152: Please see my comment on L142 above.

- L154: It was not readily clear to me what the authors meant by "early" here. It seems as if this refers to the use of the original MinION device, as opposed to the Mk1. Thus, I suggest to clarify this point.

- L161: "Read annotation of 5-mers" reads strangely to me. Based on my understanding, the reads were assigned to a specific taxon and for each read using BLASTN, the 5-mer frequency profiles were computed, and then visualized. I would thus suggest rephrasing this sentence.

- L164: It is nice to see that an unsupervised approach was used to inspect whether meaningful cluster structures would be apparent despite the high sequence error rates. However, PCA is probably not the most sensitive approach to this, albeit it is a commonly used approach for the purpose of dimension

reduction. I would thus suggest to use approaches which were developed more recently, e.g., based on Emergent Self-Organizing Maps (ESOMs) or based on Barnes-Hut Stochastic Neighbor Embedding (BH-SNE). The latter is integrated into VizBin, which (full disclosure) I am the first author of. While I did not have the per-read taxonomic assignments readily available (only the BLASTN output), I performed my own experiment and used VizBin with the FASTA files of the "equal" and the "rare" datasets. In both cases, distinct clusters with less overlap than in the PCA plot were revealed. While this could be quantitatively evaluted using a 2D clustering approach, this is likely beyond the scope of this work. Moreover, from my personal experience, PCA plots for k-mer frequency profiles are likely to be much less informative if the number of clusters increases, while ESOM- or BH-SNE-based approaches have been shown to readily resolve cluster structures even for higher numbers of clusters. I would thus suggest to elaborate on this point and would be happy to provide further information to the authors if required.

- L170: It seems that a verb is missing here, maybe "mapped" or "assigned"?

- L255: The use of "extra-species sequences" sounded a bit confusing to me in this context. I would consider linkers/primers/adapters as "extra-species", but not symbionts, parasites, or pathogens. Thus this should be clarified.

- L258-260: I found this sentence difficult to read. Please consider rephrasing it.

- L290-291: While I would also intuitively suspect more reference sequences for E. coli than for R. sphaeroides, I would suggest to provide concrete numbers here, e.g., the number of genomes for the respective species at the NCBI Taxonomy database.

- L302-303: While I am also optimistic about further technological improvements in the field of long read sequencing, I found the last part of the sentence a bit to strong and would suggest toning the last part down or removing it as it is rather speculative.

- L340: In my copy of the manuscript, there was apparently an encoding error with the "25 ?L of Elution Buffer". This is however hopefully fixed in a later version.

- L357: Same problem as with L340.

- L368: A stated above, the difference in the results between MG-RAST-based analyses and BLASTN/Kraken-based analyses could be discussed more. Here, i.e., in the Methods section, more details about the parameters used to run the various tools should be provided. Maybe, if not already planned, putting the analysis code (scripts, parameters, etc.) online would be good too.

- L380: Please include references to the panels (A and B) into the legend.

- L395: Please clarify what "combined reads" and "pass reads" refer to.

- Table 1: Please include some description as to why the runtime varies. Was it because the run simply ended, because the input material was completely consumed, or was the run simply terminated because of sufficient sequence data generated? Was any of the runs followed after a previous run, or were fresh flow cells used for each experiment? Please check the consistency of the capitalization in the table's headings.

- Table 2: The legend refers to genus-level assignments, yet the main text refers to the species level. Please clarify this point.

- Table 3: The font was different from all the other tables. This is likely to be fixed in a later version.

- Table 4: Please check the consistency of the capitalization in the table's headings, i.e., "species" vs. "Genus"

Methods

Are the methods appropriate to the aims of the study, are they well described, and are necessary controls included? Yes

Conclusions

Are the conclusions adequately supported by the data shown? Yes

Reporting Standards

Does the manuscript adhere to the journal's guidelines on minimum standards of reporting? Yes

Statistics

Are you able to assess all statistics in the manuscript, including the appropriateness of statistical tests used? There are no statistics in the manuscript.

Quality of Written English

Please indicate the quality of language in the manuscript: Acceptable

Declaration of Competing Interests

Please complete a declaration of competing interests, considering the following questions:

- Have you in the past five years received reimbursements, fees, funding, or salary from an organisation that may in any way gain or lose financially from the publication of this manuscript, either now or in the future?
- Do you hold any stocks or shares in an organisation that may in any way gain or lose financially from the publication of this manuscript, either now or in the future?

- Do you hold or are you currently applying for any patents relating to the content of the manuscript?
- Have you received reimbursements, fees, funding, or salary from an organization that holds or has applied for patents relating to the content of the manuscript?
- Do you have any other financial competing interests?
- Do you have any non-financial competing interests in relation to this paper?

If you can answer no to all of the above, write 'I declare that I have no competing interests' below. If your reply is yes to any, please give details below.

I declare that I have no competing interests.

I agree to the open peer review policy of the journal. I understand that my name will be included on my report to the authors and, if the manuscript is accepted for publication, my named report including any attachments I upload will be posted on the website along with the authors' responses. I agree for my report to be made available under an Open Access Creative Commons CC-BY license (http://creativecommons.org/licenses/by/4.0/). I understand that any comments which I do not wish to be included in my named report can be included as confidential comments to the editors, which will not be published.

I agree to the open peer review policy of the journal

To further support our reviewers, we have joined with Publons, where you can gain additional credit to further highlight your hard work (see: https://publons.com/journal/530/gigascience). On publication of this paper, your review will be automatically added to Publons, you can then choose whether or not to claim your Publons credit. I understand this statement.

Yes
