# Supplementary material for: MinION™ nanopore sequencing of environmental metagenomes: a synthetic approach
Source: Gigascience. 2017 Feb 24;6(3):1–10. doi: 10.1093/gigascience/gix007 (PMC5467020; doi:10.1093/gigascience/gix007)
Supplement: Reviewer_1_Report_(Revision_1).pdf [file gix007_Reviewer_1_Report_(Revision_1).pdf]

Reviewer Report

Title: "MinION™ nanopore sequencing of environmental metagenomes: a synthetic approach"

Version: Revision 1 **Date:** 1/16/2017

Reviewer name: Cedric Laczny, Ph.D.

Reviewer Comments to Author:

The manuscript has been thoroughly revised.

While the BLASTN analyses have been been replaced by One Codex-based analyses, I still think that the manuscript would have benefited from the use of approaches which are more robust/sensitive to long-read data, e.g, BLASR or DALIGNER, especially as the manuscript reports on the fundamental applicability/usefulness of nanopore-based metagenomic sequencing.

As qualified in the manuscript, more sensitive approaches typically also mean longer runtimes which can be a critical factor, for some areas at least, e.g., diagnosis.

Moreover, due to the fast-paced developments in this field, in particular the increase in sequencing accuracy, I understand the authors' decision.

As data accession identifiers are provided, interested peers can access the data and perform their own experiments, e.g, benchmarking more alignment approaches.

In summary, this work represented and does represent an interesting resource for the research community.

Only a few, minor comments (listed below) remain in the revised document.

- L119: "newer tools aimed at long-read data including". While these tools have been shown to work for long-read data, I would not necessarily support that they are "aimed" at this kind of data. Hence, I suggest to qualify this.

- L154: extra period after "taxa", s. "taxa. (Table 3)."

- L178 - 179: I would suggest to maybe add something like "distinct per-species clusters in the PCA plots", as this sentence reads a bit confusing to me in its current form.

- L232 - 233: "despite the resulting chimerism" reads to me like chimeras would be highly frequent, kind of dominating the contigs, after assembly. While, chimeric contigs are indeed a problem, this sentence could be qualified, e.g., "despite the potential for chimeric contig formation".

- L265 - 266: It seems only fair to also highlight the higher rate of Kraken vs. One Codex for the equal mixture with the version 5 chemistry (97.6% vs 87.4%), even though they are comparable (actually One Codex is slightly more accurate) for this mixture when using version 6 of the chemistry.

Methods

Are the methods appropriate to the aims of the study, are they well described, and are necessary controls included? Yes

Conclusions

Are the conclusions adequately supported by the data shown? Yes

Reporting Standards

Does the manuscript adhere to the journal's guidelines on minimum standards of reporting? Yes

Statistics

Are you able to assess all statistics in the manuscript, including the appropriateness of statistical tests used? There are no statistics in the manuscript.

Quality of Written English

Please indicate the quality of language in the manuscript: Acceptable

Declaration of Competing Interests

Please complete a declaration of competing interests, considering the following questions:

- Have you in the past five years received reimbursements, fees, funding, or salary from an organisation that may in any way gain or lose financially from the publication of this manuscript, either now or in the future?
- Do you hold any stocks or shares in an organisation that may in any way gain or lose financially from the publication of this manuscript, either now or in the future?
- Do you hold or are you currently applying for any patents relating to the content of the manuscript?
- Have you received reimbursements, fees, funding, or salary from an organization that holds or has applied for patents relating to the content of the manuscript?
- Do you have any other financial competing interests?
- Do you have any non-financial competing interests in relation to this paper?

If you can answer no to all of the above, write 'I declare that I have no competing interests' below. If your reply is yes to any, please give details below.

I declare that I have no competing interests

I agree to the open peer review policy of the journal. I understand that my name will be included on my report to the authors and, if the manuscript is accepted for publication, my named report including any attachments I upload will be posted on the website along with the authors' responses. I agree for my report to be made available under an Open Access Creative Commons CC-BY license (http://creativecommons.org/licenses/by/4.0/). I understand that any comments which I do not wish to be included in my named report can be included as confidential comments to the editors, which will not be published.

I agree to the open peer review policy of the journal

To further support our reviewers, we have joined with Publons, where you can gain additional credit to further highlight your hard work (see: https://publons.com/journal/530/gigascience). On publication of this paper, your review will be automatically added to Publons, you can then choose whether or not to claim your Publons credit. I understand this statement.

Yes
