# Supplementary material for: MinION™ nanopore sequencing of environmental metagenomes: a synthetic approach
Source: Gigascience. 2017 Feb 24;6(3):1–10. doi: 10.1093/gigascience/gix007 (PMC5467020; doi:10.1093/gigascience/gix007)
Supplement: Reviewer_2_Report_(Original_Submission).pdf [file gix007_Reviewer_2_Report_(Original_Submission).pdf]

Reviewer Report

Title: "MinION™ nanopore sequencing of environmental metagenomes: a synthetic approach"

Version: Original Submission **Date:** 10/1/2016

Reviewer name: Arwyn Edwards

Reviewer Comments to Author:

The manuscript presents a benchmarking exercise to evaluate the use of the MinION based nanopore DNA sequencing platform to conduct shotgun metagenomics. The authors test MinION's ability to correctly assign reads from single and mixed species sequencing runs as well as a contrived/mock community. A particular strength of the manuscript is the comparison of different analysis approaches (MGRAST, Kraken, BLAST) which might be used to analyse nanopore metagenomes, rather than just one approach. I find their overall conclusions to be reasonable and well supported by their data. The manuscript is well written, and the experimental approaches suitable for the most part.

Within the manuscript there are some issues which need to be addressed. Principally these pertain to technical issues pertaining to the 20 species mock community sequenced.

1. The authors detail (L329) that the limited quantity of mock community DNA necessitated Phi29 preamplification to obtain 1 ug DNA for library preparation. Multiple displacement amplification presents a well-known bias in the analysis of low biomass samples by shotgun metagenomics, and in some cases Phi29 amplification can itself produce chimeric products (reviewed: Binga et al. 2008 ISMEJ doi:10.1038/ismej.2008.10). Considering the authors make a considerable virtue of the reduced potential for biases introduced via chimeric assemblies (L99) and amplification-based metagenomics (L59) presented by nanopore shotgun metagenomics this presents two sources of bias which are not well detailed within the manuscript. In a revision I believe the authors could justify their use of this particular mock community better, and discuss the potential errors introduced by the Phi29 amplification in the context of their evaluation of the mock community. While I am of the opinion that the generation of an ad hoc community from the admixture of higher concentration genomic DNAs would be a fairer test of the platform than this approach, considering a potential application in low-biomass / low complexity communities identified by the authors (L300) perhaps this approach is justified. Regardless, this segment of the manuscript would benefit from clearer justification and caveating of the experimental approach.

2. Table 4 specifies both rRNA operon copy number and pg/uL DNA content. I would hope the latter reflects the actual genomic content of individual species within the mock community, but it is unclear.

As the authors are undoubtedly aware, rRNA operon copy number varies between taxa. The manuscript does not specify if/how this variation has been controlled for, given that the mock community has been staggered by rRNA operon copy number, rather than total genome content. This should be clarified, and if unaddressed, corrected for in the calculations of actual species proportions within the mock community.

3. Handling obsolecence gracefully. By the time of submission R7 flow cells, sequencing chemistry and scripts outlined in this manuscript are no longer available, and as the authors highlight, the prospect of improved performance enhancing the potential for nanopore metagenomics is approaching. Nevertheless I believe the manuscript retains considerable value in setting out a baseline in nanopore metagenomics. In a revision, I believe the manuscript would benefit from clearly acknowledging the rapid advancement within the field in the interim, and presenting the caveat that the limitations described are likely to be surpassed in the near future, if not already. This might be achieved by expanding and updating the discussion at L308-312.

4. L145: "to closely related cyanobacteria genera, Sphingobacteriaceae" In what way is the family Sphingobacteriaceae within the Bacteroidetes phylum a closely related genus to the genus Microcystis within the phylum Cyanobacteria? This is a significant taxonomic error which renders the justification presented for mis-assignment of reads open to considerable question. In a revision this statement should be corrected and the underlying cause clearly requires further thought.

Arwyn Edwards

Aberystwyth University

Methods

Are the methods appropriate to the aims of the study, are they well described, and are necessary controls included? Yes

Conclusions

Are the conclusions adequately supported by the data shown? Yes

Reporting Standards

Does the manuscript adhere to the journal's guidelines on minimum standards of reporting? Yes

Statistics

Are you able to assess all statistics in the manuscript, including the appropriateness of statistical tests used? Yes, and I have assessed the statistics in my report.

Quality of Written English

Please indicate the quality of language in the manuscript: Acceptable

Declaration of Competing Interests

Please complete a declaration of competing interests, considering the following questions:

- Have you in the past five years received reimbursements, fees, funding, or salary from an organisation that may in any way gain or lose financially from the publication of this manuscript, either now or in the future?
- Do you hold any stocks or shares in an organisation that may in any way gain or lose financially from the publication of this manuscript, either now or in the future?
- Do you hold or are you currently applying for any patents relating to the content of the manuscript?
- Have you received reimbursements, fees, funding, or salary from an organization that holds or has applied for patents relating to the content of the manuscript?
- Do you have any other financial competing interests?
- Do you have any non-financial competing interests in relation to this paper?

If you can answer no to all of the above, write 'I declare that I have no competing interests' below. If your reply is yes to any, please give details below.

Declaration: My team is currently working on a manuscript focused upon the use of nanopore sequencing for in-field metagenomics. See http://biorxiv.org/content/early/2016/09/07/073965. Given the contrasting focus and objectives of each manuscript I do not consider the overlap to be sufficient to present a non-financial competing interest likely to affect an objective evaluation of the manuscript; I have no other competing interests.

I agree to the open peer review policy of the journal. I understand that my name will be included on my report to the authors and, if the manuscript is accepted for publication, my named report including any attachments I upload will be posted on the website along with the authors' responses. I agree for my report to be made available under an Open Access Creative Commons CC-BY license (http://creativecommons.org/licenses/by/4.0/). I understand that any comments which I do not wish to be included in my named report can be included as confidential comments to the editors, which will not be published.

I agree to the open peer review policy of the journal

To further support our reviewers, we have joined with Publons, where you can gain additional credit to further highlight your hard work (see: https://publons.com/journal/530/gigascience). On publication of

this paper, your review will be automatically added to Publons, you can then choose whether or not to claim your Publons credit. I understand this statement.

Yes
