# Supplementary material for: MinION™ nanopore sequencing of environmental metagenomes: a synthetic approach
Source: Gigascience. 2017 Feb 24;6(3):1–10. doi: 10.1093/gigascience/gix007 (PMC5467020; doi:10.1093/gigascience/gix007)
Supplement: Reviewer_3_Report_(original_submission).pdf [file gix007_Reviewer_3_Report_(original_submission).pdf]

Reviewer Report

Title: "MinION™ nanopore sequencing of environmental metagenomes: a synthetic approach"

Version: Original Submission **Date:** 10/3/2016

Reviewer name: Son Hoang Nguyen

Reviewer Comments to Author:

The authors conducted a study of using MinION nanopore sequencing to characterize environment metagenomes. The 2D long reads were used in attempt to assign taxa of different synthetic mixtures up to 20 species. Several analytical tools was applied for this purpose.

The manuscript is scientifically sound and basically well-written. The results show potential application of nanopore sequencing in the field of environment metagenomics, although the performances are still limited for the time beings.

There are some opinions from my point of view:

1. In Table 1, the unit for the fourth column (Total bp) must be Mbp instead of Gbp when it comes to the yield of a typical MinION run.

2. Table 2 is quite difficult to follow and catch the point. I expect a graph for better visualization instead of numeric table in this circumstance.

In addition, there is a significant deviation between results of using MG-RAST with the other two but I hardly found any discussion about this.

It is also better if the authors could briefly review the 3 methods used in their study for community analysis and the reasons they were chosen in a bit more details.

3. The authors claimed that using long reads has advantages over short read data for metagenomics studying. If applicable, a real comparison of such that case, e.g. using MinION vs Illumina data for community analysis, would make the point more convincing.

Methods

Are the methods appropriate to the aims of the study, are they well described, and are necessary controls included? Yes

Conclusions

Are the conclusions adequately supported by the data shown? Yes

Reporting Standards

Does the manuscript adhere to the journal's guidelines on minimum standards of reporting? Yes

Statistics

Are you able to assess all statistics in the manuscript, including the appropriateness of statistical tests used? Yes, and I have assessed the statistics in my report.

Quality of Written English

Please indicate the quality of language in the manuscript: Acceptable

Declaration of Competing Interests

Please complete a declaration of competing interests, considering the following questions:

- Have you in the past five years received reimbursements, fees, funding, or salary from an organisation that may in any way gain or lose financially from the publication of this manuscript, either now or in the future?
- Do you hold any stocks or shares in an organisation that may in any way gain or lose financially from the publication of this manuscript, either now or in the future?
- Do you hold or are you currently applying for any patents relating to the content of the manuscript?
- Have you received reimbursements, fees, funding, or salary from an organization that holds or has applied for patents relating to the content of the manuscript?
- Do you have any other financial competing interests?
- Do you have any non-financial competing interests in relation to this paper?

If you can answer no to all of the above, write 'I declare that I have no competing interests' below. If your reply is yes to any, please give details below.

I declare that I have no competing interests

I agree to the open peer review policy of the journal. I understand that my name will be included on my report to the authors and, if the manuscript is accepted for publication, my named report including any attachments I upload will be posted on the website along with the authors' responses. I agree for my report to be made available under an Open Access Creative Commons CC-BY license

(http://creativecommons.org/licenses/by/4.0/). I understand that any comments which I do not wish to be included in my named report can be included as confidential comments to the editors, which will not be published.

I agree to the open peer review policy of the journal

To further support our reviewers, we have joined with Publons, where you can gain additional credit to further highlight your hard work (see: https://publons.com/journal/530/gigascience). On publication of this paper, your review will be automatically added to Publons, you can then choose whether or not to claim your Publons credit. I understand this statement.

Yes
