# Supplementary material for: MinION™ nanopore sequencing of environmental metagenomes: a synthetic approach
Source: Gigascience. 2017 Feb 24;6(3):1–10. doi: 10.1093/gigascience/gix007 (PMC5467020; doi:10.1093/gigascience/gix007)
Supplement: Reviewer_4_Report_(Original_Submission).pdf [file gix007_Reviewer_4_Report_(Original_Submission).pdf]

Reviewer Report

Title: "MinION™ nanopore sequencing of environmental metagenomes: a synthetic approach"Original Submission

Version: Original Submission **Date:** 10/11/2016

Reviewer name: Alfonso Benitez-Paez, Ph.D.

Reviewer Comments to Author:

The authors report the study of three synthetic microbial communities using WGS in the portable DNA sequencer, MinION, as well as genome analysis of single species merged in such artificial communities. Although genome sequencing analysis can be found regularly in the scientific literature, the authors put special attention to the usage of MinION reads to perform environmental metagenomics, an approach of limited study by using third generation sequencing technologies, therefore this study compiles data and analyses of interest for future approaches regarding the better description of microbial communities in terms of species inventory and function as well. Common microbial community taxonomy and diversity analysis were used to evaluate the data produced by MinION device concluding a modest performance of MinION device.

In my opinion, this work represent an interesting workflow to help in the achievement of valuable information regarding the complete assessment of environmental microbial communities, where the limitations in terms of read length of the second generation of sequencing technologies constitute major issues concerning the species identification. As a consequence, I recommend this manuscript for publication in GigaScience journal once the following changes have been incorporated to the main text and supporting material:

Major concerns:

1. The authors claim in the introduction that "very short reads can lack information needed to properly identify the source of the read due to conservation of gene sequences across related organisms"; and given that in line 142 they describe that DNA reads obtained from single-species runs range from 5bp to hundreds of kbp, I miss a filtering step on MinION data to retain larger fragments (e.g. > 1 or >2 kb) previous to the taxonomy assessment. This could improve the results exhibited in the Table 2.

2. Why the authors did not consider to use the "low-input" kit from ONT to perform the sequencing analysis of the mock staggered community?

3. In line 154 the authors describe use of MinION device but two lines below describe to using the MkI device. Have the authors observed any difference in the performance of these both devices? Could this change of device affect in somehow the analysis?

4. In my opinion, the very low amount of 2D reads retrieved from the sequencing process is the major handicap of this technology to assess metagenomics from environmental- or human-derived samples (median of ~5% of all runs). The authors have to be more conclusive in this way and clearly state that unless performance in terms of number of reads be extremely increased, the MinION platform would not be appropriate to perform metagenomics of complex samples.

5. The author must discuss in a deeply manner the findings regarding the annotation of metagenomic sequences derived from MinION. It is quite evidently that MG-RAST did not process the MinION data in a proper way and the algorithms behind every assignment tool should be compared to get consensus procedure that better fit to MinION data.

6. Data coming from the analysis of the 20-species mock staggered community are disappointing and interesting at the same time. In one hand, WIMP seems to work very well in terms of the performance needed for metagenomics analyses. Given that WIMP directly connect to NCBI database, it is able to compare MinION data with one of the largest DNA repositories offering reliable results at real-time regarding the composition of the microbial community under study. On the other hand, the strong bias at microbial composition level observed when the mock staggered community was analyzed could be also explained by the genome structure itself. The author should correlate the bias among the observed and expected composition of the microbial community for every single species with the GC content or other parameter of genome complexity. Additionally, the authors should measure the absolute abundance of certain species at the original mock staggered community and after the linear amplification with Phi29 polymerase by qPCR methods. This will be very helpful to shed light on the origin of such abundance bias.

Methods

Are the methods appropriate to the aims of the study, are they well described, and are necessary controls included? Yes

Conclusions

Are the conclusions adequately supported by the data shown? Yes

Reporting Standards

Does the manuscript adhere to the journal’s guidelines on minimum standards of reporting? Yes

Statistics

Are you able to assess all statistics in the manuscript, including the appropriateness of statistical tests used? There are no statistics in the manuscript.

Quality of Written English

Please indicate the quality of language in the manuscript: Choose an item.

Declaration of Competing Interests

Please complete a declaration of competing interests, considering the following questions:

- Have you in the past five years received reimbursements, fees, funding, or salary from an organisation that may in any way gain or lose financially from the publication of this manuscript, either now or in the future?
- Do you hold any stocks or shares in an organisation that may in any way gain or lose financially from the publication of this manuscript, either now or in the future?
- Do you hold or are you currently applying for any patents relating to the content of the manuscript?
- Have you received reimbursements, fees, funding, or salary from an organization that holds or has applied for patents relating to the content of the manuscript?
- Do you have any other financial competing interests?
- Do you have any non-financial competing interests in relation to this paper?

If you can answer no to all of the above, write 'I declare that I have no competing interests' below. If your reply is yes to any, please give details below.

I declare that I have no competing interests

I agree to the open peer review policy of the journal. I understand that my name will be included on my report to the authors and, if the manuscript is accepted for publication, my named report including any attachments I upload will be posted on the website along with the authors' responses. I agree for my report to be made available under an Open Access Creative Commons CC-BY license (http://creativecommons.org/licenses/by/4.0/). I understand that any comments which I do not wish to be included in my named report can be included as confidential comments to the editors, which will not be published.

I agree to the open peer review policy of the journal

To further support our reviewers, we have joined with Publons, where you can gain additional credit to further highlight your hard work (see: https://publons.com/journal/530/gigascience). On publication of this paper, your review will be automatically added to Publons, you can then choose whether or not to claim your Publons credit. I understand this statement.

Yes
